# Secondary Structure across the Bacterial Transcriptome Reveals Versatile Roles in mRNA Regulation and Function

**DOI:** 10.1371/journal.pgen.1005613

**Published:** 2015-10-23

**Authors:** Cristian Del Campo, Alexander Bartholomäus, Ivan Fedyunin, Zoya Ignatova

**Affiliations:** 1 Biochemistry, Institute of Biochemistry and Biology, University of Potsdam, Potsdam, Germany; 2 Biochemistry and Molecular Biology, Department of Chemistry and Biochemistry, University of Hamburg, Hamburg, Germany; Consejo Superior de Investigaciones Científicas, SPAIN

## Abstract

Messenger RNA acts as an informational molecule between DNA and translating ribosomes. Emerging evidence places mRNA in central cellular processes beyond its major function as informational entity. Although individual examples show that specific structural features of mRNA regulate translation and transcript stability, their role and function throughout the bacterial transcriptome remains unknown. Combining three sequencing approaches to provide a high resolution view of global mRNA secondary structure, translation efficiency and mRNA abundance, we unraveled structural features in *E*. *coli* mRNA with implications in translation and mRNA degradation. A poorly structured site upstream of the coding sequence serves as an additional unspecific binding site of the ribosomes and the degree of its secondary structure propensity negatively correlates with gene expression. Secondary structures within coding sequences are highly dynamic and influence translation only within a very small subset of positions. A secondary structure upstream of the stop codon is enriched in genes terminated by UAA codon with likely implications in translation termination. The global analysis further substantiates a common recognition signature of RNase E to initiate endonucleolytic cleavage. This work determines for the first time the *E*. *coli* RNA structurome, highlighting the contribution of mRNA secondary structure as a direct effector of a variety of processes, including translation and mRNA degradation.

## Author Summary

Messenger RNA (mRNA) is intrinsically prone to form higher order structures which is optimized for mRNA stability in the cell. We took advantage of recent developments in high throughput sequencing technologies and coupled them with RNA structure-probing approaches to provide a high resolution view of the mRNA secondary structure of *Escherichia coli* on a global, transcriptome-wide scale. Our data highlight the contribution of mRNA secondary structure as a direct effector of a variety of processes, including translation initiation and termination, mRNA abundance and degradation. This goes beyond the primary function of mRNA as an information entity in the transfer of the genetic information and places it more centrally in regulating fidelity of translation.

## Introduction

The primary role of mRNA in cellular physiology is to act as an informational molecule for translating ribosomes. Yet, emerging evidence places mRNA more centrally in regulating biogenesis of the encoded protein, including cotranslational folding and insertion and interactions with auxiliary factors [1–3]. mRNA is intrinsically prone to form higher order structures, i.e. secondary and tertiary folded motifs. RNA structures tend to be highly dynamic and undergo conformational changes on a microsecond time scale [4]. Furthermore, one linear single-stranded RNA sequence can potentially adopt several differently complex secondary (such as hairpins and stem-loops) and tertiary folds (i.e. stabilized by interactions between distantly located sequences).

Recent developments in high throughput sequencing technologies and their coupling with RNA structure-probing approaches provided a comprehensive map of the secondary structure of the whole cellular transcriptome of yeast, plants and metazoans [5–11] and highlight the broad contribution of RNA structure to modulating gene expression. Conceptually, the mRNA structure is determined by probing its susceptibility to enzymatic cleavage (nucleases S1, P1 for single stranded and RNaseV1 for double-stranded regions), or chemical modifications (e.g. 2’-hydroxyl alkylation of exposed A, G, C or U with 1-methyl-7-nitrosatoic anhydride (1M7), methylation of exposed N1 of adenines or N3 of cytosines by dimethyl sulfate (DMS), modification of exposed N3 of uridines and to smaller extent of N1 of guanines with 1-cyclohexyl-(2-morpholinoethyl)carbodiimide metho-p-toluene sulfonate) (reviewed in [12]). Global *in vitro* analysis of the total mRNA of yeast subjected to either single-strand or double-strand enzymatic digestion revealed that coding regions exhibit higher propensity to be involved in secondary structure than non-coding regions [6]. Further *in vivo* analysis using cell-permeable DMS to probe unpaired A and U argues that on a global level, in rapidly dividing yeast and mammalian cells, the mRNA secondary structure of the coding sequences (CDSs) does not impede translation elongation, highlighting the role of RNA-binding proteins and ATP-dependent helicases in modulating the mRNA dynamics *in vivo* [8]. Only few mRNA structures that are selected for regulatory purposes persist [8,13,14]. Importantly, probing all four nucleotides with 1M7 (icSHAPE) in mouse embryonic stem cells reveals that some persistent structural elements are similar in *in vivo* and *in vitro* [9,15]. Moreover, the *in vitro* folding landscape of an mRNA does not differ from that *in vivo*, but the exchange between adjacent structures *in vivo* is much faster than *in vitro* [16]. Here we combine the power of three different sequencing technologies, parallel analysis of the mRNA structure (PARS), ribosome profiling and RNA-seq to extract, to our knowledge for a first time, the structural features in mRNA selected for regulation of gene expression in *Escherichia coli*.

In bacteria, based on available single gene examples (summarized in [17]), it has been axiomatically assumed that secondary structure propensity correlates with mRNA stability which in turn is proportional to mRNA abundance and translatability. However, microarray-based analysis of more than 2000 genes in *E*. *coli* shows that computed secondary structure stability is not predictive of increased mRNA abundance [17]. Even highly-translated mRNAs with high abundance can be very unstable [17]. Furthermore, detailed single gene studies have shown the significant influence of tRNA abundance or mRNA secondary structure as key modulators of translation elongation rate [18–20]. Interestingly, in regions with high propensity to secondary structure, codons pairing to high-abundance tRNAs, i.e. translated faster [20],are preferentially selected; secondary structure and fast translating codons act in an opposing manner on translational speed, potentially cancelling out their individual effects and smoothing overall translational speed [21]. In physiological conditions, initiation is rather rate-limiting [22] and initiation rate is affected largely by mRNA sequence features [23]. Reduced folding of codons 3’ adjacent to the start codon enhances expression [24,25].

To address the impact of mRNA secondary structure across the *E*. *coli* transcriptome, we used parallel analysis of RNA structure (PARS) coupled to deep-sequencing [6], which reports on the intrinsic propensity of protein (ribosome)-free mRNA to partition in secondary structures. We exploited PARS to select candidate sites for regulatory RNA structures. We then complemented PARS with ribosome profiling [26] and RNA-Seq [27] to determine the impact of RNA structure on translation efficiency and mRNA abundance in the cell, respectively. With this combined approach, we uncovered structural elements that may facilitate different steps of translation. The recognition site of RNase E, a major player in mRNA decay in *E*. *coli*, was also inferred from the PARS analysis. Our global analysis corroborates early reports from single-gene studies [28–30] and features on a global level the common recognition signature of RNase E cleavage, which is composed of double-stranded and single-stranded segments. More broadly, our study provides a comprehensive foundation for understanding the impact of mRNA secondary structure in bacterial gene expression with implications in design and engineering of synthetic genes.

## Results

### PARS reveals globally conserved structural features among *E*. *coli* transcripts

**Fig 1:**
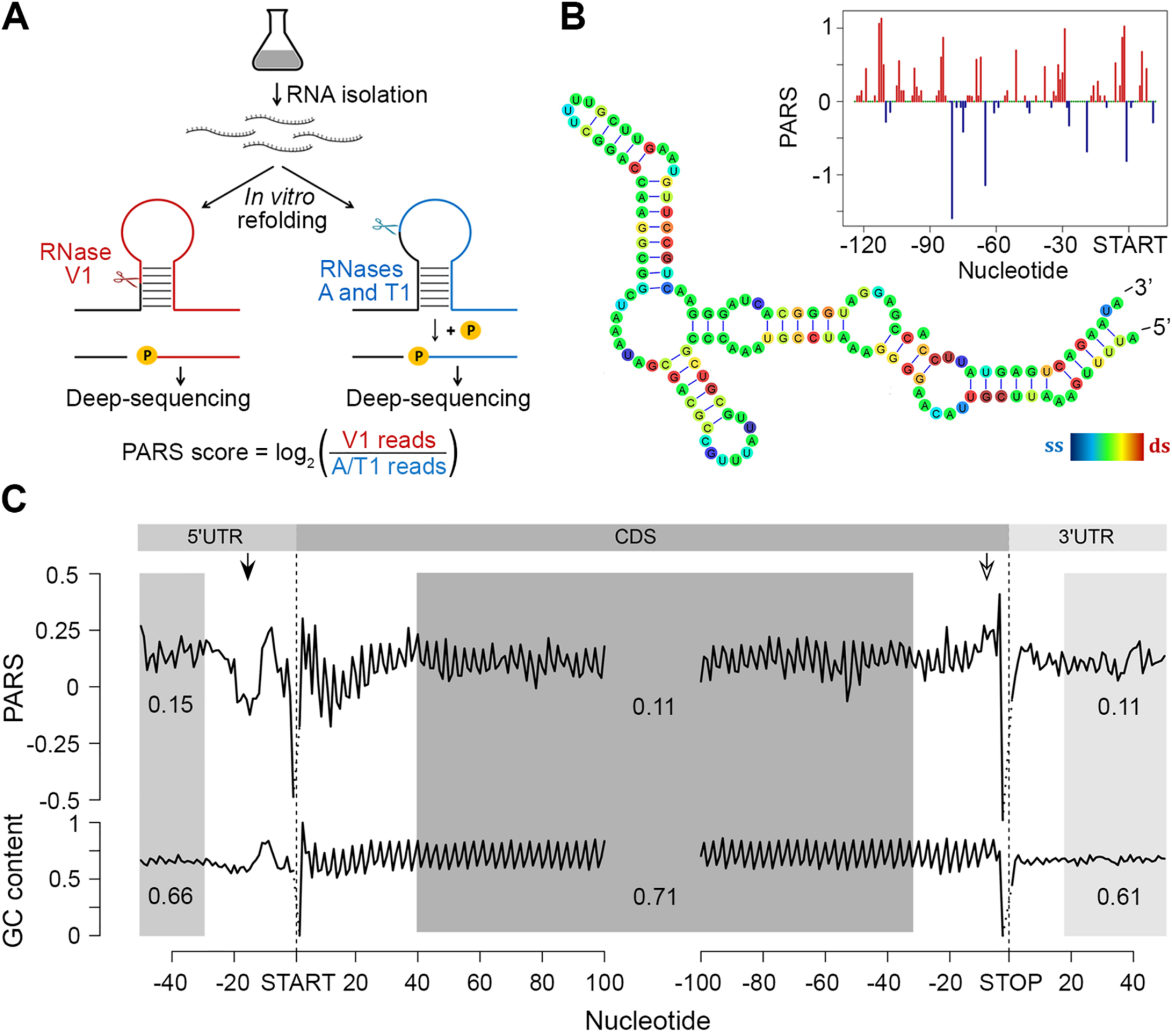
PARS analysis. (A) Overview of modified PARS approach. RNase V1 cleaves double-stranded RNA and combination of RNases A/T1 the single stranded RNA with optimal activities at physiological pH (7.0). RNAse A/T1 usage requires an additional phosphorylation step prior to library generation. (B) The PARS score of the *rpoS* leader sequence (inset) was overlaid with the experimentally determined structure [64]. Double-stranded nucleotides with positive PARS score are colored red, single-stranded nucleotides with negative PARS score–blue, nucleotides with missing PARS score or equal to zero–green. The color intensity of the *rpoS* nucleotides reflects the PARS scores (rainbow legend). (C) Metagene analysis of protein-coding transcripts. Average PARS score for each nucleotide (top) and GC content (bottom) across the 5’UTRs, CDS and 3’UTRs of all protein-coding transcripts, aligned at the start or stop codon, respectively. For the shaded areas the average PARS scores or GC content is calculated; thus note the deviations from the total GC content of 51% in *E*. *coli*. Unstructured region upstream of the start codon and structured sequence preceding the stop codon are marked by arrows with filled and open arrow heads, respectively.

To assess the intrinsic propensity of the *E*. *coli* transcriptome to partition in secondary structures, we isolated total mRNA (i.e. in absence of proteins and ribosomes) from exponentially growing *E*. *coli* culture and subjected it to PARS with some modifications of the original protocol [6] (Fig 1A; details are provided in the Methods section). The total mRNA was either digested with single strand-specific RNase A/T1 or with double strand-specific RNase V1 (Fig 1A) and coupled to a massively parallel sequencing to depth of ~50 million reads (~16 million uniquely mappable reads per sample). RNase T1 and RNase A cleave specifically at unpaired guanosine and pyrimidines (cytosine and uracil), respectively, while RNase V1 cleaves at all four paired bases. The results are highly reproducible across replicates (Pearson correlation coefficients *R* = 0.96 and *R* = 0.95 for V1 and A/T1 digestions, respectively, S1 Fig). The PARS score was calculated for each nucleotide, which also exhibits good reproducibility on transcriptome-wide and single-transcript levels (S1C and S1D Fig), and a positive PARS score indicates preferential involvement in double-stranded structure (Fig 1A and 1B). At a selected threshold of 1.0 [6] for reliable reads at each position (S1 Fig), we obtained structural information for ~900,000 nucleotides covering 2,536 *E*. *coli* genes. The results from PARS are in excellent agreement with known RNA structures and match four experimentally validated RNA structures (Fig 1B; S2 Fig), including also the whole 16S rRNA. Furthermore, we performed additional independent experimental validation of the *ppiC* transcript; the PARS values recapitulate the results from orthogonal structural probing of *ppiC* (S1 Fig).

Metagene analysis of the transcripts aligned at their start and stop codons shows that *E*. *coli* CDSs have a propensity to form double-stranded structure to a level that is similar to the structure propensity of the 5’- and 3’-untranslated regions (UTRs) (Fig 1C). This global trend is different than that in eukaryotic organisms. In yeast, UTRs are less structured than CDSs [6]. Conversely, in metazoans [31] and humans [11] UTRs are, on average, more structured than coding regions. A well-defined periodic pattern is present only in the CDSs but not in the 5’ and 3’UTRs as detected by discrete Fourier transform (S3 Fig) with first nucleotide being the most structured (S3 Fig). Three nucleotide periodicity is also detected in yeast [6], *A*. *thaliana* [5], mouse [32] and human [11] and is intrinsic to the structure of the genetic code (see the periodic pattern of the GC content, Fig 1C), consistent with prior computational predictions for various genomes [33]. We noticed, however, that in some regions the mRNA structure deviates from the nucleotide content, e.g. a uniform unstructured region around 20 nt upstream of the initiation start and more structured region upstream of the termination codon (Fig 1C). These positions may provide candidate sites for functional conformation of mRNA *in vivo* and we address their role below.

The region 10–30 nt downstream of the initiation was also less structured than the average PARS score of the CDS (Fig 1C). Less structured regions at the 5’ start of the CDSs facilitate initiation and general gene expression [24,25], a trend which is also present in the human [11] but not in the yeast [6] transcriptome.

### Intrinsic secondary structure propensity of the CDS influences elongation only locally in some genes

**Fig 2:**
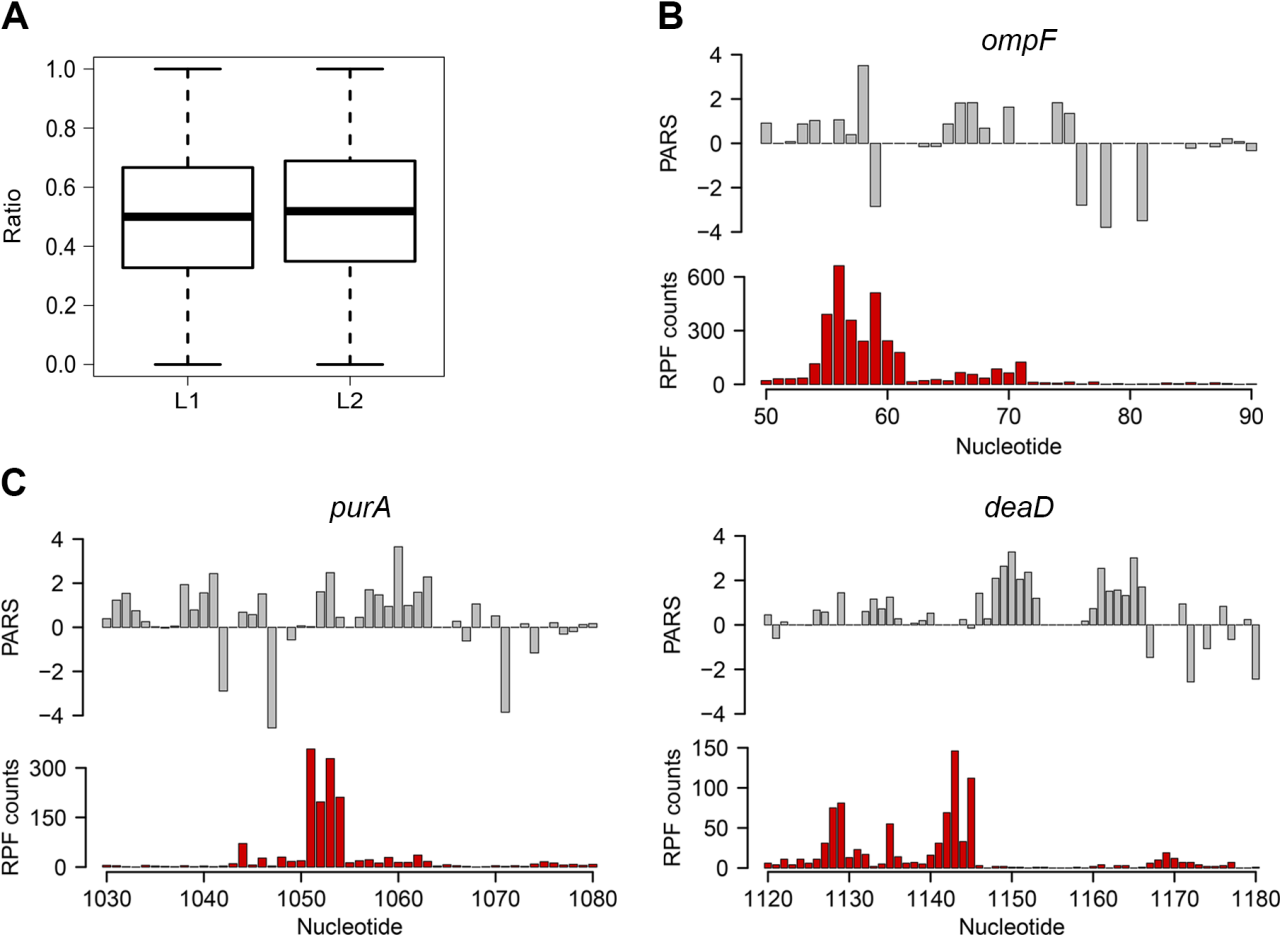
Ribosomal pausing induced by secondary structure in CDS. *(*A) Globally, ribosomal pausing is not significantly affected by the presence of secondary structure in the CDS. Box plot analysis of the ratio of RPF upstream (L1) calculated from Eq 2 and downstream (L2) calculated from Eq 3 of detected secondary structures (*P* = 0.1209, Kolmogorov-Smirnov test). (B,C) Ribosomal pausing is observed within coding sequences above the 80^th^ percentile (panel A). Examples of *ompF* transcript with previously validated secondary structure [65] (B) as well as newly detected genes (C) for which a local secondary structure causes non-uniform ribosomal distribution. Aligned PARS score (upper panel, gray) with the RPF counts (bottom panel, red) at each nucleotide.

We next asked whether the intrinsic secondary structure propensity of the CDS influences the translation (elongation) efficiency and correlates with mRNA abundance in the cell. We complemented the PARS analysis with ribosome profiling which captures the positions of translating ribosomes with nucleotide resolution [26] which showed high reproducibility between biological replicates on a global (S4 Fig) and single gene level (S1 Fig). We hypothesized that a persisting mRNA structure would induce ribosomal pausing which would be detected by enrichment of ribosome-protected fragments (RPFs) upstream of an mRNA structured stretch. A structured stretch was defined when 6 nt within a window of 10 nt show a positive PARS score (for details see Methods section and S5 Fig). In total, within the CDSs we extracted 908 stretches with high structure propensity *in vitro*. For the majority of the structured stretches we did not detect an accumulation of the RPF upstream of them (Figs 2A and S5) suggesting that the majority of these structures may not persist *in vivo* and do not influence the elongating ribosomes that is consistent with the observation in yeast and mammalian cells [8]. Nonetheless, a sizeable fraction of structured sites in the CDS (above the 80^th^ percentile, 87 positions) caused ribosomal pausing, i.e. L_1_>L_2_ (Eqs 2 and 3; Fig 2A). Along with the genes with previously validated structures (Fig 2B and S1 Table), our analysis revealed some promising candidates for novel functional RNA structures (Fig 2C; S1 Table). One of the genes, *deaD*, encodes a DEAD-box RNA helicase that functions in large ribosomal subunit assembly [34] and RNA degradation under cold shock [35]. Contrary to the prevailing views for DeaD function at only low temperature, recent evidence describes its expression over a broad temperature range but with large variation in expression level [36]. It is tempting to speculate that the newly identified persistent structure in *deaD* (Fig 2C) may regulate its expression level at different temperatures through a structure-induced translational pausing.

Slow-translated regions, mostly formed by clustering of suboptimal codons, are enriched in *E*. *coli* membrane proteins at the beginning of their transmembrane domains [37]. Similarly to yeast, these regions may promote interaction with the signal recognition particle [2] and thus facilitate membrane targeting and translocation. Since a large fraction of the identified structural sites that correlated with accumulated RPF reads were in membrane proteins (S1 Table), we analyzed the distance between the pausing positions and start of the transmembrane domains. The majority of the pausing sites were within 11 to 80 amino acids downstream of the membrane domains (S5 Fig). Strikingly, this distance interval closely resembles the 30–72 amino acid span needed to exit the ribosomal tunnel [38]. Thus, secondary structure-induced ribosome stalling may play a role in membrane targeting in a manner similar to the transient pausing of translation by suboptimal codons [2,37].

### mRNA abundance correlates with the mean structural propensity of the coding sequence

**Fig 3:**
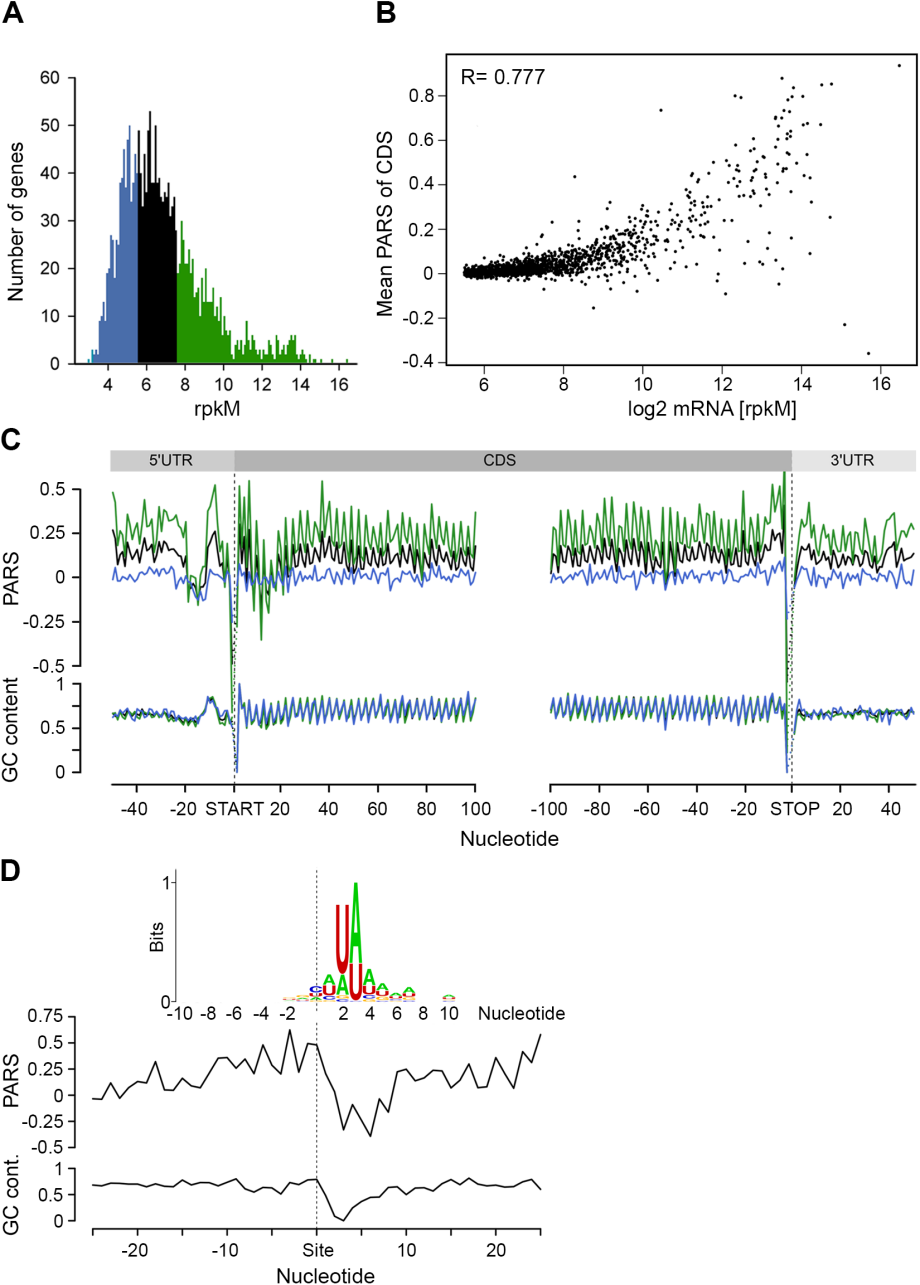
mRNA structure correlates with mRNA abundance. (A) Distribution of transcript abundance, expressed in gene read counts normalized by the length of CDS per kilobase and the total mapped reads per million (rpkM). The 30% least (blue) and most (green) abundant genes from the reliably detected genes (S4 Fig) are highlighted. (B) Dependence of the mean PARS score on the mRNA abundance of the middle (black) and most (green) abundant transcripts as defined in panel A. R = 0.777, Pearson correlation coefficient. (C) Average PARS score (top) and GC content (bottom) for each position of all transcripts (black curve) as well as the 30% most (green) and least (blue) abundant. (D) Average PARS score (top) and GC content (bottom) for each position around the top 64 RNase E cleavage sites (S2 Table). Inset, the sequence logo of the aligned RNase E cleavage sites, spanning from -10 to +10 nt.

Clearly, under physiological conditions, the secondary structure propensity of the majority of CDSs had no impact on the elongating ribosomes. However, mRNA structure is important for a variety of processes, including maintenance of stability and half-life [39]. To quantify the transcriptome, we performed an RNA-Seq experiment [27] which exhibited high reproducibility between biological replicates (S4 Fig). Comparison of the mean PARS score over the CDS revealed a clear correlation with the mRNA abundance (Fig 3A and 3B): the 30% most abundant transcripts exhibited higher secondary structure than the 30% least abundant genes (*p* = 2.2*10^−16^, Mann-Whitney test, Fig 3C). Thus, we next asked whether low abundance transcripts are more susceptible to degradation. In *E*. *coli*, RNase E is a key enzyme in RNA metabolism and has a major influence on the mRNA life cycle [40]. Recent RNA-Seq-based analysis identified ~1,800 RNase E target sites within *E*. *coli* mRNAs [41]. Within the genes with a transcript load over the threshold of 1.0 (S1E Fig), we identified 64 RNase E cleavage positions (Fig 3D, S2 Table) which score among the first 100 cleavage sites [41]. However, those genes did not cluster within the gene group with the lowest abundance and lowest propensity to form secondary structure.

The cleavage site of RNase E is at an unpaired sequence [41] which lacks a specific sequence motif but is rather enriched in A and U (Fig 3D, inset). Single gene studies propose the importance of stem-loop structures 5’ adjacent to the A/U rich target sites of RNase E [28–30]. Strikingly, we observed this common signature for the 64 identified RNaseE target sites: the unpaired target region is preceded by a structured mRNA stretch (Fig 3D). Also, this structural signature is common for all additional ~1,800 RNase E target sites. Furthermore, we analyzed the structural features of additional endonucleases which have been identified under RNase E-depleted conditions [41]. The target sites of other endonucleases bears no secondary structure upstream the cleavage site and thus significantly differ than that of RNase E (S6 Fig) implying that the structural signature of the RNase E target sites is of importance for its recognition. Notably, the target sites of all endonucleases lack a specific consensus sequence motif but are rather enriched in specific nucleotides (S6 Fig). This observation is consistent with mutational study of the unpaired RNase E cleavage site, which suggests that RNase E cleavage is affected by the extent of A and U rather than their order [29].

### Unstructured sequence upstream of the start codon is a general feature of *E*. *coli* genes

**Fig 4:**
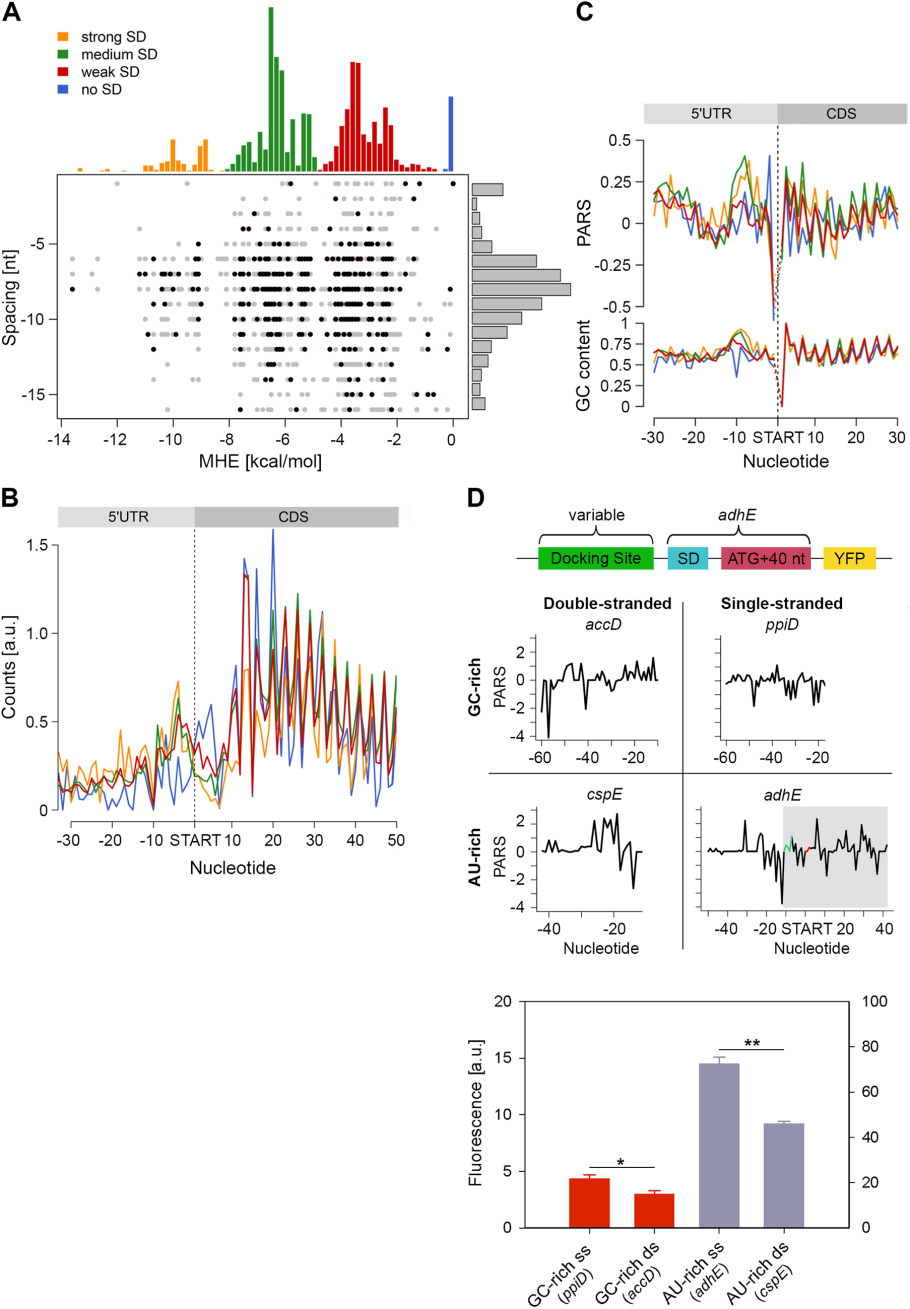
Stronger SD sequence has a higher propensity to form secondary structure which does not correlate with the translation efficiency. (A) SD strength does not correlate with translation efficiency (i.e. the total RPFs per coding mRNA) of a gene. SD hybridization energies fall into four major distributions: strong SD, MHE < -8.5 kcal/mol; medium SD, -8.5 < MHE < -4.4 kcal/mol; weak SD, -4.4 < MHE < -2 kcal/mol; no SD, MHE > -2.0 kcal/mol. For each gene (dot) the MHE (horizontal axis) of the SD sequence is plotted against SD spacing (vertical axis), defined as the distance between the second to last nucleotide of the SD and the start codon. Genes with the 30% highest ribosomal density are highlighted as black dots. (B) Cumulative plots of ribosomal density for all genes grouped by SD strength. Genes were aligned by the first nucleotide of the start codon. (C) Average PARS score smoothed over 3 nt (top) and GC content (bottom) for each position of the four SD strength classes, aligned by the start codon. The four different SD groups are color coded as in panel A. (D) FACS expression analysis of *adhE* whose original docking site was replaced by three other docking sites with clearly different sequence (AU-rich or GC-rich) and different PARS score. Only the sequence upstream of the SD (green on the PARS profiles) was replaced. The common part of *adhE* which is fused to YFP (schematic inset) is shadowed on the PARS profiles. The average PARS score over the docking site (12 to 30 nt upstream of the start codon, red on the PARS profiles are): *adhE*–-0.564, *ppiD*–-0.495, *cspE*–0.724, *accD*–0.665. Data are means (n = 3) ± standard error of the mean (s.e.m.). *, *P* <0.05; **, *P* <0.01.

We detected a unique structural feature for the *E*. *coli* transcripts which is not present in yeast and human [6,11]: the region 7–12 nt upstream of the start codon is significantly more structured (mean value 0.17) than the average CDS (mean value 0.11, Fig 1C, marked with an arrow). A large fraction of genes in *E*. *coli* is initiated by Shine-Dalgarno (SD) sequence upstream of the start codon and its hybridization strength to the anti-SD of the 16S rRNA (3’-UCCUCCAC-5’) determines initiation fidelity. We computed the minimum hybridization free energy (MHE) between the anti-SD sequence and genes whose translation was initiated by SD which revealed four major groups (referred to as strong, medium, weak, and no SD groups, Fig 4A). [The complete list of all parameters plotted in Fig 4A is available on our webpage (http://www.chemie.uni-hamburg.de/bc/ignatova/tools-and-algorithms.html)]. A randomized sample of the same size displayed different MHE distribution (S7 Fig), implying the functional importance of different SD groups. Moreover, the four groups that are selected based on the strength of the SD:anti-SD pairing resemble previous definitions (which however use a threshold of *MHE* value of -4.4 kcal/mol to select for more stringent SD sequences) [42]. Note that we did not use any threshold and also included SDs with lower MHE (weak SD) that occur naturally, e.g., AAGG [43] with MHE of −2.9 kcal/mol.

In general, the GC content of each SD group mirrored the SD strength. SD:anti-SD base pairing is crucial to align the P-site of the ribosome on the start codon, hence the optimal spacing between the SD and the start codon is 7–8 nt [44,45] which we also detected independent of the strength of the SD (Fig 4A). To our surprise, we did not observe any correlation between SD strength and translation efficiency, which was determined by the density of ribosomes (RPF) per mRNA (Pearson correlation, *R* = 0.03, Fig 4A). Highly translated genes did not preferably cluster in any of the SD groups (Chi-square test: *p* = 0.3539, black symbols, Fig 4A). Notably, even some genes lacking an SD sequence were also highly translated (Fig 4A). We also noticed that for genes with strong and medium SD more RPFs accumulated in the SD vicinity (Fig 4B); these genes were slightly more structured in the SD vicinity than genes with weak SD or those lacking an SD, which is however mirrored in the GC content in this region (Fig 4C).

By analyzing the profiles of the gene groups with different SD strength, we noticed one striking feature: the region starting at ~20 nt upstream of the start codon is the most unstructured region within each gene (mean value of -0.06 for the region -22 to -13 nt, Fig 1C). Strikingly, this feature is not recapitulated by the GC content suggesting that it is not selected through A/U-rich sequences and may play active role in regulating translation initiation. Clearly, ribosomes attach to this unstructured site since we detected reads in the ribosome profiling data set at this location (Fig 4B). The ribosome binds in a biphasic-kinetics mode to some mRNAs and both phases have clear implications for the expression of the corresponding gene [46]. While the second transition in the kinetic curves represents the positioning of the anti-SD of 16S rRNA over the SD sequence, the role of first phase is unclear [46]. Usually multiphasic transitions suggest multiple binding events, thus we hypothesized that this unpaired region might represent an additional unspecific binding site of the 30S to facilitate its positioning over the SD. To examine the physiological importance of this unpaired site in expression of the encoded protein, we compared four different sites: AU-rich sequences with low (i.e. unstructured) and high (i.e. structured) PARS score and GC-rich sequences with low and high PARS score. Each site was fused to the first 50 nt of *adhE* (SD and first 42 nt of the CDS) upstream of the YFP. The resulting expression was quantified by flow cytometry (schematic in Fig 4D). Notably, constructs with less structured upstream regions resulted in higher expression than their more structured counterparts with similar sequence content (compare AU-rich with single- and double-stranded docking site—*adhE* vs *cspE*, or GC-rich with single- and double-stranded docking site—*ppiD* vs *accD*; Fig 4D). The variant with unpaired AU-rich region exhibited higher expression than the one with unpaired GC-rich sequence (compare *adhE* and *ppiD*, Fig 4D). In general, AU-rich single-stranded regions are less structured than GC-rich single-stranded regions, which correlates with the mean PARS score over this region (-30 –-12 nt upstream of the start codon); the mean PARS score of unpaired AU-rich *adhE* is -0.564 and of the GC-rich *ppiD* is -0.495 (Fig 4D). The *adhE* gene exhibited the highest expression, which might be argued that it due to using part of *adhE* as an invariable backbone in our constructs (schematic Fig 4D). To exclude this argument, we replaced the invariable *adhE* part with a fragment of the same size originating from *ppiD* (SD and first 42 nt of the CDS, S7 Fig). Replacing the original *ppiD* region upstream of the SD with the most unstructured sequence of *adhE* enhanced the expression by twofold (S7 Fig).

In sum, our results feature the poorly structured region at ~20 nt upstream of the start codon as an additional binding site of the ribosome distinct from SD binding, and its secondary structure propensity correlates with the expression of the downstream CDS.

### Higher secondary structure upstream of the stop codon has a likely role in termination

**Fig 5:**
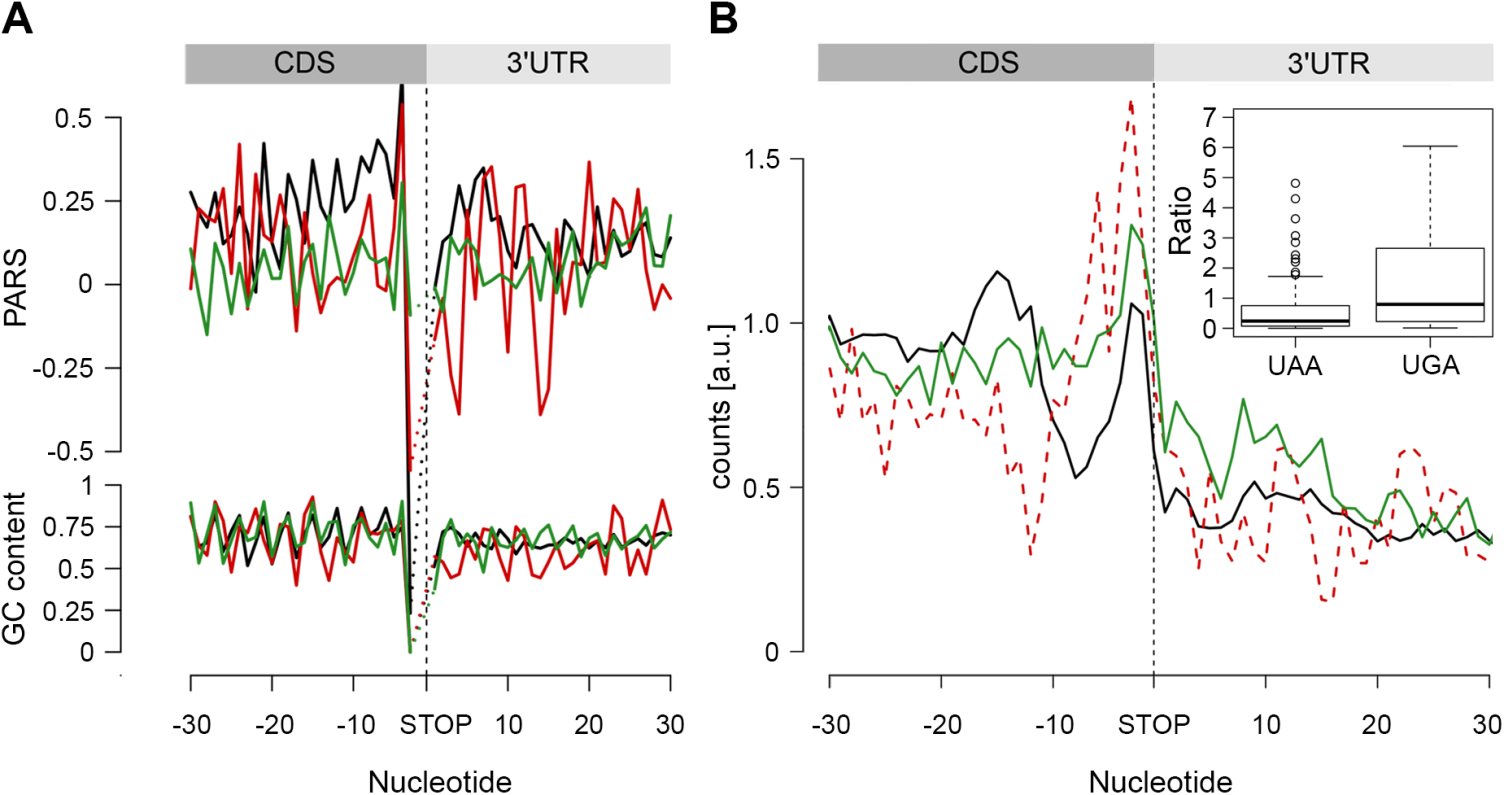
The stop codon of operon genes is more structured than non-operon genes. (A) Average PARS score and GC content for each position of genes terminating with UAA (black), UAG (red) and UGA (green) stop codons. (B) RPF coverage around the stop codon region for genes terminated by UAA (black), UAG (dashed red) and UGA (green) stop codons. Only genes with coverage over 60 reads (S4D Fig) were used; overlapping operon genes were excluded. Note, that UAG-terminated genes are included only for comparison; their low number prevents performing any statistical analysis. The inset shows, for both UAA- and UAG-terminated genes, the ratio between the RPFs downstream of the stop codon (3 to27 nt) and a mean of the CDS. The readthrough value for the majority of the genes was zero; only genes with a value higher than zero are plotted.

In the metagenome analysis we noticed that the region upstream of the stop codon is more structured than the average PARS score of the CDS and 3’-UTR, whereas a GC content of this region does not significantly differ from the average CG content of the CDS (Fig 1C). Genes terminated with the UAA codon exhibited the highest propensity to form secondary structures in the 3’-termini of the CDS (*p* = 2.2*10^−16^, Mann-Whitney test, Fig 5A). Notably, we observed an enrichment of RPF reads ~10–30 nt upstream of the UAA-termination codon (*p* = 6.94*10^−6^, Mann-Whitney test) suggesting a persistent secondary structure (Fig 5B).

In *E*. *coli*, a large fraction (53%) of protein-coding genes is organized as polycistronic mRNAs in operons to facilitate the association and physical interactions of functionally related proteins. The SD sequence of an overlapping or a closely positioned downstream gene (S8 Fig) may influence our analysis, resulting in an apparent higher structure in the 3’ vicinity of the upstream gene. Thus, we next separately analyzed the secondary structure upstream of the stop codon of protein-coding genes organized in operons from those in non-operons; the operon group is additionally divided in two groups: non-overlapping, with a distance of ≥ 30 nt from the downstream gene, and overlapping, with a downstream gene located < 30 nt to the upstream gene. Only UAA-terminated genes showed increased PARS score (*p* = 0.00023 for non-overlapping, *p* = 3.2*10^−10^ for overlapping, *p* = 4.07*10^−5^ for non-operon, Mann-Whitney test, S8 Fig) in the 3’ vicinity of the coding sequence and this feature is not mirrored by the GC content. Also, the frequency of the three stop codons (UAA, UAG and UGA) is similar for all gene groups and resembles stop codon usage in the genome (S8 Fig).

We hypothesized that secondary structure upstream of the stop codon may influence the termination fidelity of the UAA-terminated genes. Additional in-frame stop codons may act as safeguards against leaky termination. We reasoned that if the structure in the vicinity of the UAA stop codon influences termination, those genes would show lower frequency of ribosomes in the 3’-UTR. We analyzed the ribosome occupancy downstream of the UAA- and UGA-terminated genes (considering it in general as a readthrough). Overlapping genes were excluded from this analysis as ribosomes terminating the upstream gene cannot be unambiguously distinguished from ribosomes initiating the downstream gene. Strikingly, we observed a low but significant fraction of RPF reads downstream of the UGA stop codon while RPF reads in the 3’ UTR of the UAA-terminated genes were nearly not detectable (Fig 5B). This phenomenon occurred in the background of a similar distribution of additional in-frame stop codons downstream of all terminating codons: UAA–10.7%, UGA–8.7% and UAG–7.4%. Together, this analysis suggests that structure upstream of the stop codon may enhance the termination fidelity of the UAA-codon terminated genes.

## Discussion

We provide a comprehensive analysis of the intrinsic structure propensity of the *E*. *coli* mRNAome, which combined with physiological analysis, identifies structural features implicated in the regulation of translation efficiency in *E*. *coli*. These include a universal unstructured site at ~20 nt upstream of the start codon, which we postulate to serve as a non-specific docking of the 30S ribosomal subunit; this site differs from the SD:anti-SD binding site. Within the CDSs, we identified a small set of persisting structured regions that transiently stall the ribosomes and may regulate protein integration into the membrane. On a global level, however, the secondary structure of the CDS has no effect on translation elongation *in vivo*, highlighting the importance of energy-dependent processes (for example ATP-dependent helicases, ribosomes) or passive elements (for example single-stranded RNA binding proteins) in regulating mRNA structures in the cell [8]. Moreover, the propensity of CDSs to form secondary structure is counterbalanced by selection of codons that pair to high-abundance tRNAs which in general smooths the overall translation speed [21]. For the majority of *E*. *coli* transcripts, translation is initiated by complementation of the anti-SD of the 30S subunit with the SD sequence upstream of the start codon. Our analysis reveals that SD sequences are often occluded in secondary structures with a highly dynamic reversible folding/unfolding kinetics on a microsecond time scale [4,47]. Thermodynamically, for an anti-SD to outcompete such a secondary structure the 30S subunit needs to be already in the close vicinity of the SD. Although in the current analysis neither ribosome profiling nor PARS analysis bear kinetic information or can reveal a sequence of binding events, we envision that the unfolded site upstream of the SD sequence may act as a primary unspecific docking site of the 30S subunit to enable interactions with the SD sequence within its unfolding window. Supportive for our model is the observed biphasic kinetics of ribosome binding to some mRNAs with an unclear first phase and a second owing to anti-SD:SD interactions [46]. Also, current approaches to predict translational rates based only on SD strength fail to accurately account for known differences in translation initiation rates [48]. Our expression analysis convincingly shows that the unstructured site at ~20 nt influences translation of the downstream CDS and the expression level correlates with the degree of its unfolding. The global genome-wide analysis features this unstructured region upstream of the start codon as the most unfolded structure in the *E*. *coli* genome but its size seems smaller than the 30S subunit (Fig 1C). The contacts with the mRNA might be established by the essential S1 protein, which is the only ribosomal protein with an mRNA-binding affinity. Furthermore, S1 protein, which is essential for unfolding of structured SD [49], attaches to mRNA 11 nt upstream of the SD [50] which is approximately the position of the unpaired region.

We also observed an enrichment of ribosomes upstream of a persistent secondary structure which is found ~4–8 nt 5’-adjacent of the UAA stop codon. Previous research on termination regulation provides appropriate context for the interpretation of these results. The efficiency of translation termination (or conversely, the rate of termination suppression) is sensitive to the 5’ and 3’ sequence in immediate proximity of the stop codon [51]. Moreover, the nature of the corresponding codon (i.e. nucleotides 4–6) upstream of the stop codon plays an important role in the efficiency of termination [52]. Systematic exchange of different codons prior the stop codon evidence the highest termination efficiency by those encoding bulky amino acids, in the absence of a broader sequence motif. Interactions of the bulky residues of the nascent peptide with the ribosomal tunnel are suggested to slow down terminating ribosome prior to termination which enhances the termination fidelity [52]. The accurate positioning of the A-site over the stop codon determines the accuracy in termination and suppresses readthrough: A-rich sequences preceding the stop codons distort the ribosomes in the P-site which alters the stop-codon decoding in the A-site [53]. In comparison, our analysis features a persistent mRNA secondary structure upstream of the UAA stop codon which is not encoded by a universal sequence motif but is similarly responsible for a ribosomal slowdown. By drawing an analogy to these studies, we suggest that the secondary structure upstream of the UAA stop codon slows down the elongating ribosome which may assist the accurate positioning of the ribosomal A-site for accurate decoding of the UAA stop codon.

Another striking aspect of our analysis is the identification of a global signature of RNase E cleavage site. Earlier single-gene studies proposed the importance of secondary structures 5’ upstream of the single-stranded cleavage site [28–30,54]. Our analysis corroborates those observations and features a structured region upstream of the A/U rich unpaired site as common signature of RNase E cleavage sites on a transcriptome-wide scale. This signature can be reconciled with the RNase E crystal structure: while a single-stranded segment only fits in the shallow channel leading to the RNase E active site [54], the internal flexibility of the quaternary structure [55] can clearly accommodate secondary mRNA structures. The latter significantly shortens the distance between the cleavage site and 5’ terminus and may explain how distant 5’ termini of the mRNA facilitate catalysis [54].

In summary, our approach of structurally probing bacterial mRNA *in vitro* with PARS, complemented with RNA-Seq and ribosome profiling, reveals structural features of importance for a variety of cellular processes. Although coding mRNA sequences show a frequent intrinsic propensity to form secondary structure, only a small fraction influences translation fidelity *in vivo*. Our combined approach features the importance of applying a variety of techniques to unambiguously evaluate structure-function relationships in physiological context.

## Methods

### RNA structural probing by deep sequencing

The *E*. *coli* MC4100 strain was cultured at 37°C to mid-log phase (OD_600_ ~ 0.4) in LB media. Total RNA was extracted using TRIzol reagent (Invitrogen) and the sample was enriched in mRNA by depleting small RNAs with GeneJET RNA Purification Kit (Fermentas) and ribosomal RNA with two cycle of MICROBExpress Bacterial mRNA Enrichment Kit (Ambion) which reduces the amount of rRNA to appr. 25% of the total sequencing reads. To probe the RNA structure, two μg of enriched mRNA were resuspended in 45 μl of DEPC water and denatured for 3 min at 95°C, refolded at 37°C, combined with 10x RNA-structure buffer with pH 7.0 (100 mM Tris, 1 M KCl, 100 mM MgCl_2_) and digested for 1 min at 37°C with either 0.05 U RNase V1 (Life Technologies) or a combination of 2 μg RNase A and 5 U RNase T1 (Thermo Scientific). The reaction was stopped by extracting the RNA with phenol-chlorophorm. RNases A/T1 were preferred as they exhibit a stable activity at pH 7.0 [56] compared to nuclease S1 which has a pH optimum ~5.0 and aberrant activity at pH 7.0. At pH 7.0, high concentrations of nuclease S1 are required; however at such high concentrations S1 also digests double-stranded regions [57]. The nucleolytic reaction was stopped by extracting the RNA with phenol-chlorophorm. The RNase A/T1-digested sample was phosphorylated with T4 PNK (NEB) and purified with RNA Clean & Concentrator kit (Zymo Research). Both the V1 and A/T1 digested samples were randomly fragmented in buffer with pH 9.2 (100 mM Na_2_CO_3_, 2 mM EDTA) for 12 min at 95°C. The reaction was stopped by adding 560 μl 300 mM NaOAc, pH 5.5, followed by isopropanol precipitation. RNA size selection and generation of the cDNA libraries were performed as described [26].

### Ribosome profiling

To isolate mRNA-bound ribosome complexes and extract the RPFs we used a previously described approach [58] with some modifications. For the isolation of RPFs, an aliquot of 100 A_260_ units of ribosome-bound mRNA fraction (prior to ultracentrifugation in the sucrose gradients) was subjected to nucleolytic digestion with 10 units/μl micrococcal nuclease (Fermentas) for 10 min at room temperature in buffer with pH 9.2 (10 mM Tris pH 11 containing 50 mM NH_4_Cl, 10 mM MgCl_2_, 0.2% triton X-100, 100 μg/ml chloramphenicol and 20 mM CaCl_2_). The monosomal fraction was separated by sucrose density gradient (15–50% w/v). The total RNA was isolated from monosomes using the hot SDS/phenol method. Micrococcal nuclease also cleaved rRNA into fragments with a size similar to the RPFs. The sample was enriched predominantly in one rRNA fragment which was removed by subtractive hybridization at 70^0^ C using a 5’-biotin-5’-GCCTCGTCATCACGCCTCAGCC-3’. DNA oligonucleotide along with μMACS Streptavidin Kit (Myltenyi Biotec) to remove the biotin-labeled DNA/rRNA hybrids. Both randomly fragmented mRNAs and RPFs extracted from monosomes were denatured for 2 min at 80°C, and 3’-dephosphorylated with T4 polynucleotide kinase (NEB) for 90 min at 37°C in the corresponding buffer without ATP (NEB). RNA was precipitated by standard methods. Subsequently, 20-35-nt RNA fragments were size selected on a denaturing 15% polyacrylamide gel stained with SYBR Green II (Invitrogen) using 10-100-nt leader (Affymetrix) as a standard. The gel was extracted, precipitated and resuspended in DEPC water.

### Random mRNA fragmentation and cDNA libraries

To generate the RNA-Seq sample to which the ribosome profiling data are compared, 20 μl of the enriched mRNA (as described above) was mixed with equal volume of 2x alkaline fragmentation solution (2 mM EDTA and 100 mM Na_2_CO_3_ pH 9.2) and incubated for 40 min at 95°C. The reaction was stopped by adding 560 μl 300 mM NaOAc pH 5.5, followed by isopropanol precipitation. The optimal time for fragmentation of mRNA was determined using GAPDH mRNA (0.25 μg; Fermentas) and the spectra were recorded with BioAnalyzer (Agilent RNA 6000 Kit).

The cDNA libraries from RPFs and fragmented mRNAs were prepared using a modified protocol for miRNA [59] which yielded much higher resolution and allowed for calculation of the position of the ribosomes with codon precision (S4 Fig). Gel-purified RNA fragments were dissolved in 10 mM Tris pH 8.0 and used for the preparation of the cDNA library via direct adapter ligation [59] including some additional steps. As both mRNA fragments and RPFs were hydroxylated at their 5’- and 3’-termini, after the ligation of the adapter to the 3’-end, the fragments were 5’-phosphorylated with T4 polynucleotide kinase in ATP-containing buffer (NEB) for 30 min at 37°C followed by the adaptor ligation at the 5’-termini. The fragments with adaptors at both termini were size selected on a denaturing 15% polyacrylamide gel, extracted and reverse transcribed with RevertAid H Minus Reverse Transcriptase (Fermentas) using 5’-CAAGCAGAAGACGGCATACGA-3’ primer and PCR-amplified with *Pfu* DNA Polymerase (Fermentas) for 10 to 20 cycles. The PCR amplified DNA library was quantified with BioAnalyzer (Agilent DNA 1000 Kit) and sequenced on the Illumina GAIIx platform.

### Mapping of the sequencing reads

Sequenced reads were quality trimmed using *fastx-toolkit* (0.0.13.2; quality threshold: 20) and sequencing adapters were cut using *cutadapt* (1.2.1; minimal overlap: 1 nt) discarding reads shorter than 12 nucleotides. Processed reads were mapped to the *E*. *coli* genome (strain MG1655, version U00096.2, downloaded from NCBI) using Bowtie (0.12.9) allowing a maximum of two mismatches for the RNA-Seq and ribosome profiling data and a maximum of three mismatches for the PARS data. Strain MC4100 is a derivative of MG1655 with four major deletions [60]

The number of raw reads unambiguously aligned to ORFs in both RNA-Seq and ribosome profiling data sets, from two biological and one technical replicates were used to generate gene read counts, by counting the number of reads whose middle nucleotide (for even read length the nucleotide 5' of the mid-position) fell in the CDS. Gene read counts were normalized by the length of the unique CDS per kilobase (rpkM) and the total mapped reads per million (rpM) [27]. In this mapping round, reads aligning to rRNA and tRNA genes were excluded since a large fraction of them map non-uniquely due to the multiple copies of those genes. Mapping of 5S and 16S RNA was done separately allowing no mismatches to only one copy of the rRNA reference sequence.

### Computing the PARS score

The first nucleotide of the mapped reads from V1 or A/T1 digested samples, each derived from two biological replicates, was assigned to a nucleotide position in the genome and the counts were normalized to the sequencing depth. For each position, we computed the PARS score which is defined as the log_2_ of the ratio between the number of reads per million (rpM) from the V1-treated and the A/T1-treated samples (to each we added a small number 1, to avoid division by zero and to reduce the potential overestimating of low-coverage bases [6]). RNase A hydrolyzes at single-stranded C and U nucleotides and RNase T1 at single-stranded G nucleotides, thus we excluded all adenines from the analyses. In addition, zero PARS score may result at positions with the same count values for A/T1 and V1 digestion, which are usually located in regions with highly flexible structure. As a minimum PARS coverage per transcript we used a threshold of 1.0 per transcript length (S1 Fig) termed transcript load [6] which is defined as the sum of combined PARS readouts of the biological replicates per transcript divided by the effective transcript length (that is the annotated transcript length minus the number of unmappable nucleotides); the same threshold was used in yeast PARS analysis named as load of a transcript [6]. For the cumulative plots, all genes were aligned either to the start or the stop codon and for each position the mean of the PARS score of the two biological replicates was calculated. The GC content was calculated considering only the non-zero PARS score entries.

Periodicity of average PARS score in the CDSs and 5’UTR and 3’UTR was analyzed by Discrete Fourier transform (S3 Fig). The following regions were analyzed: over 10 to 99 nt downstream of the start codon, 99 to10 nt upstream of the stop codon (i.e. excluding possible influences of the initiation and termination codons but keeping the translation reading frame) for the CDSs, and 50 to 11 nt upstream of the start codon or downstream of the stop codon for the 5’UTR and 3’UTR, respectively. The periodicity for each of the three nucleotides in a codon was calculated also over the same region of the CDSs (S3 Fig).

### Modeling the sampling error between biological replicates

To select a reliable minimum of read counts per gene and to assess the influence of counting noise, we computed the binomial partitioning of total counts between two independent biological replicates [26] of the RNA-Seq and ribosome profiling from bacteria grown in LB. Genes were binned logarithmically based on the total number of their reads. The standard deviation of the ratio (repl#1/(repl#1 + repl#2)) across each bin was computed as a function of the mean sum of reads in each bin. In addition, a constant variance was added to the theoretical predictions accounting for other sources of error, yielding:

$$\sqrt{\frac{p(1-p)}{n}+s^{2}}$$ (1)

where *p* represents the probability to assign a read to replicate #1, *n* is the total number of sequencing reads from replicate #1 and replicate #2 and *s* was obtained by fitting Eq 1 to the data (S4 Fig).

### Detection of RPF enrichment upstream of secondary structures

To determine positions whose secondary structure may influence elongation we used two approaches: CDS were systematically screened for double-stranded stretches (1) with a window of 10 nt containing 4 to 8 structured nt (i.e. with positive PARS score), or (2) using the mean PARS score within a window with different size (10 or 20 nt) (S5 Fig). A 10-nt-window with 6 structured nt delivered the best result considering the number of the selected positions (908 positions, S5 Fig) and was chosen in the analysis.

To define RPF enrichment upstream of a selected secondary structure (L_1_), the RPF counts over 29 nt upstream of the double-stranded stretch (RPF1) were compared to the RPF counts over 29 nt (1st-30th nt) downstream (L_2_) of the detected stretch (RPF2). Read counts were normalized by the total number of reads for the whole region [61]:

$$L_{1}=\frac{RPF_{1}}{RPF_{1}+RPF_{2}}$$ (2)

$$L_{2}=\frac{RPF_{2}}{RPF_{1}+RPF_{2}}$$ (3)

### Determination of codon periodicity in the RPF and RNA-Seq data sets

Reads with length of 23–25 nt which were unambiguously mapped to the 1000 most expressed genes were combined for the RNA-Seq or ribosome profiling and binned by their length. To compute the codon periodicity in the RNA-Seq and ribosome profiling data sets, we used the reads mapped to the 3’-ends of the corresponding ORFs which were positioned at one of the three stop codons (UAG, UAA and UGA).

### Detection of SD sequences

For all annotated genes, the MHE was calculated between sequences 1–25 nt upstream of the start codon and anti-SD sequence (3’-UCCUCCAC-5’) using *RNAsubopt* (2.1.5; default parameters) from the *Vienna RNA Package* [62]. For each 8mer, the calculated MHE was assigned to the 8^th^ base as described [63] and the minimum of the calculated MHE of all 8mers was taken as an identifier for the SD sequence and used to determine the corresponding spacing. To designate different SD groups based on their MHE we used a randomization control. The random sample was created in two different ways: (1) by generating all possible random 8-mer sequences (65,536 sequences) or (2) by choosing each nucleotide randomly within the 8-mer (444,000 sequences). For both randomized groups we received similar results. For comparison to the natural SD, 4,400 random sequences were selected which resemble the *E*. *coli* gene number in S7 Fig.

### Footprint analysis with fluorescently-labeled mRNA

*In vitro* transcribed RNA of *ppiC* was 3’ end-labeled with 10 μM pCp-Cy3 (Jena Bioscience) using 15 U T4 Ligase 1 (NEB). 2 μg of fluorescently-labeled RNA was structure probed with 0.05 U of RNase V1 (Ambion) or with a dilution 1:7000 of combined RNase A/T1 (Thermo Scientific), in conditions identical to the PARS experiment. The digestion was stopped with phenol chlorophorm extraction, precipitated overnight at 4°C and resuspended in 10 μl of 2x RNA Loading Dye (Thermo Scientific). In parallel, a ddNTP-Sanger sequencing PCR reaction was performed using 20 pmol of a 3’-fluorescently(Cy3)-labeled primer, in the presence of 400 ng of DNA template, 10 μM dNTPs, 1.25 U Pfu DNA Polymerase (Thermo Scientific), Pfu Polymerase Buffer and 1 mM of each ddNTP. PCR was performed according to the manufacturer instructions in a volume of 15 μl. After addition of 2x RNA Loading Dye, all samples were boiled for 3 min at 95°C and loaded on a 6% PA, 1x TBE, 7M UREA gel (50x40 cm), already pre-run for 30 min at 50W. The gel was then run for 3 h at 50W and the fluorescence was detected using a fluorescent gel imager.

### Expression analysis

In each biological replicate, cells were grown in LB medium till OD_600_ = 0.5 and induced with 1 mM IPTG for 90 minutes. The median expression of the YFP-fused constructs was quantified in a population of approximately 10^5^ cells by flow cytometry on a FACSCalibur (BD Bioscience). The forward (fcs) and sideward (ssc) scatter was recorded at each measurement and the data were processed by Flowing software 2. The values were normalized to the autofluorescence background of untransformed cells transformed.

### Quantitative RT-PCR

Total mRNA was extracted using the GeneJET RNA Purification Kit (Fermentas) and treated with DNase I (Fermentas). The cDNA was synthesized with RevertAid H Minus Reverse Transcriptase (Fermentas) and quantitative RT-PCR was performed on a StepOnePlus Real-Time PCR system (Applied Biosystems) using template-specific primers. The values were normalized to the amount of the total RNA.

### Statistical analysis

All data analyses were performed with in-house algorithms in Pearl and R. Differences between the distributions were assessed for significance by a nonparametric Mann-Whitney test, and enrichment of RPF was assessed by a Kolmogorov-Smirnov test. Note that we used Mann-Whitney U test, also called Wilcoxon rank-sum test, which is suitable for unpaired data for which no normal distribution can be assumed. To determine codon periodicity, Kullback-Leibler divergence was used to measure the deviation of the observed distribution of the 3’-end of the mapped read from a uniform distribution. Differences in the expression (FACS experiments) were evaluated using two-tailed Student’s *t*-test. Differences were considered statistically significant when *P*< 0.05.

### Data access

All sequencing data files are available from Gene Express Omnibus database, GSE63817.

## Supporting Information

S1 Fig Reproducibility of the PARS results. Pearson correlation between the log_2_ of read coverage for each transcript with load >1 (see panel E) digested by RNase V1 (A) or RNases A/T1 (B) in the two biological replicates. (C) Reproducibility of the PARS score for each nucleotide. To reduce the crowding in the plot, only 200000 randomly selected nucleotides were plotted. (D) Single gene example on the reproducibility of the various sequencing data. (E) Number of transcripts as a function of the transcript load [6], i.e. the PARS readouts from the merged biological replicates divided by the effective transcript length (that is the annotated transcript length minus the number of unmappable nucleotides). A threshold of 1 (vertical dashed line) was selected as also used previously for yeast PARS data [6]. (F) Footprint analysis of fluorescently-labeled *ppiC* mRNA digested with 0.05 U (lane 1) or 0.01 U (lane 2) RNase V1 compared to undigested mRNA (lane 3). The RNase V1-digestion pattern mirrors the V1 sequencing counts. The graphic insert represents an exemplary comparison between the intensity of the bands (gray bars) from designated area from the gel (horizontal lines between 207–234 nt) and the counts for the same gene obtained from the deep sequencing of the RNase V1 digested sample. The sequence derived from the Sanger sequencing (included next to the gel) is complementary to that in the plot.
(TIF)

S2 Fig Correlation between the PARS score and transcripts with known secondary structure. The PARS score was overlaid with the determined with OmpF [65], 5S rRNA [66] and 16S rRNA structure. The color intensity of the nucleotides reflects the PARS scores (rainbow legend). For more details on the PARS-based colorcoding see the legend to Fig 1B. For 16S rRNA, PARS score was overlaid with the determined structure. Solvent exposed helices were selected from the crystal structure [67,68] and overlaid with the experimentally determined PARS values. The solvent-exposed regions are cleaved first and this first phase of nucleolysis reports on the native structure allowing for more conservative PARS analysis. Nucleotides 60–107 –helix 6; nt 116–239 –helix 7 to 10; nt 572–880 –helix 20 to 26; nt 1236–133 –helices 41 and 42; nt 1397–1542–44 and 45. The color intensity of the 16S rRNA nucleotides reflects the magnitude of the PARS scores.
(TIF)

S3 Fig Periodicity in the structure of the *E*. *coli* CDSs. (A) Discrete Fourier transform analysis. Analyses were performed with the average PARS score over 10 to 99 nt downstream of the start codon, 99 to10 nt upstream of the stop codon for the CDSs, and 50 to 11 nt upstream of the start codon or downstream of the stop codon for the 5’UTR and 3’UTR, respectively. (B) Average PARS score for each of the three nucleotides of a codon, averaged across all codons.
(TIF)

S4 Fig Reproducibility and variability analysis of the RNA-Seq and Ribo-Seq. (A, B) Reproducibility of randomly fragmented mRNAs (A) and RPFs (B) of two biological replicates. The Pearson correlation coefficients, calculated between the log_2_ of the read coverage for each transcript with counts > 60 (see panel C, D) indicate that the RiboSeq and RNA-Seq analyses are highly reproducible. (C, D) Variability analysis of counting statistics on the error in quantification of RNA-Seq (C) and ribosome profiling (D). The two independent biological and technical mRNA (A) and RPF (B) replicates were used to estimate the biological variation compared to the technical one. The technical replicates are dominated by counting noise, thus s = 0 (Eq 1). A threshold of 120 total counts (i.e., 60 counts for each replicate) was chosen as for total reads >120 the variability approached the infinite-counts asymptote and the contribution of the counting statistics was little. For the RNA-Seq data set the fitting parameters are *p* = 0.47 and s = 0.16, and for the RPF data set are *p* = 0.58 and s = 0.15. By setting a threshold to 60 reads both in mRNA-Seq and RPF-analysis, the technical error is smaller than 5% of the biological variation. In total, 1.955 genes have >60 mRNA and RPF reads and have PARS over the selected threshold of 1 (S1 Fig).
(TIF)

S5 Fig Defining structured mRNA regions within the CDSs. (A) The search is performed by varying the number of structured nucleotides (i.e. with positive PARS score) within a window of 10 nt. The numbers in brackets denote the number of 80^th^ percentile positions within the whole set of detected structured positions. (B) The search is performed using the mean PARS score within a variable window (10 or 20 nt) under the restriction that within a window at least 5 nt (5 out of 10 nt or 5 out 20 nt) or 10 nt (10 out of 20 nt) have a PARS score different than zero. Note that this approach also cannot select for a minimal threshold PARS score over which the L_1_/L_2_ ratio becomes significant. PARS score gives the propensity of each nucleotide to partition between single or double stranded structure, therefore this propensity differs from the gain of energy which is determined by the type of nucleotide, the context and other factors. (C) Distance of the last residue of a transmembrane helix and the first nucleotide of a detected secondary structure which causes ribosomal stalling. The transmembrane helices of membrane proteins with structure-induced ribosome accumulation were predicted with www.cbs.dtu.dk/services/TMHMM/. Note that for *nanT* two structured regions were detected; the upper one reports on the structured region detected at 1234 nt. aa, amino acid.
(TIF)

S6 Fig Structure of the RNase E target sites. (A) The structural signature of the RNase E target sites differ significantly from that of other endonucleases (-8 to +2 nt, *p* = 0.0066, Mann-Whitney test). Average PARS score (top) and GC content (bottom) for each position around all identified ~1,800 RNase E cleavage sites (solid line) and additional ~5000 endonucleolytic sites (dashed line) detected under RNase E-depleted conditions [41]. (B) Frequency of the nucleotides around the RNase E cleavage site or other endonucleases whose PARS plot is shown in A.
(TIF)

S7 Fig The structure propensity of the sequence upstream of SD correlates with expression. (A) Randomization of SD sequences. The MHE of paring randomized sequences (gray) with the anti-SD of the 16s rRNA is compared to the MHE distributions of naturally occurring SDs (Fig 4A). The fully randomized sample of all possible variations of randomized sequences of 8-nt length was ~65,000, however only 4,400 randomly chosen sequences (gray) are plotted to match the number of *E*. *coli* ORFs. The smoothed lines represent kernel density estimation (right y-axis). Color coding of the naturally occurring *E*. *coli* SD sequences is in Fig 4A. (B) FACS expression analysis of *ppiD* whose original sequence upstream of the SD (schematic) was replaced by that of *adhE* which has clearly different PARS score (*adhE*–-0.564, *ppiD*–-0.495). Data are means (n = 3) ± standard error of the mean (s.e.m.).**, *P* <0.01.
(TIF)

S8 Fig Stop codon distributions and secondary structure of different gene groups. (A) Examples of genes organized in operons containing overlapping genes (upper panel) or non-overlapping (bottom panel) genes. RPF counts are plotted against the nucleotide position of operons. The gray vertical lines denote the boundaries of each ORF; the distance between the ORFs is given in nt in the schematic below the RPF-coverage profile. Negative numbers denote overlapping ORFs. (B) Average PARS score and GC content around the stop codon of different gene groups terminated with UAA (black), UAG (red) and UGA (green) stop codons. (C) Frequency of the three stop codons in different gene groups. UAA (black), UGA (green) and UAG (red).
(TIF)

S1 Table List of genes with identified ribosomal stalling induced by mRNA secondary structure. Green, membrane proteins; blue, ribosomal proteins; orange, cytosolic proteins.
(DOCX)

S2 Table List of the 64 RNase E cleavage positions. Positions denote the gene coordinates in the *E*. *coli* chromosome on either the forward (fwd) or reverse (rvs) strand.
(DOCX)
