# Supplementary figures and images for: Secondary Structure across the Bacterial Transcriptome Reveals Versatile Roles in mRNA Regulation and Function
Source: PLoS Genet. 2015 Oct 23;11(10):e1005613. doi: 10.1371/journal.pgen.1005613 (PMC4619774; doi:10.1371/journal.pgen.1005613)

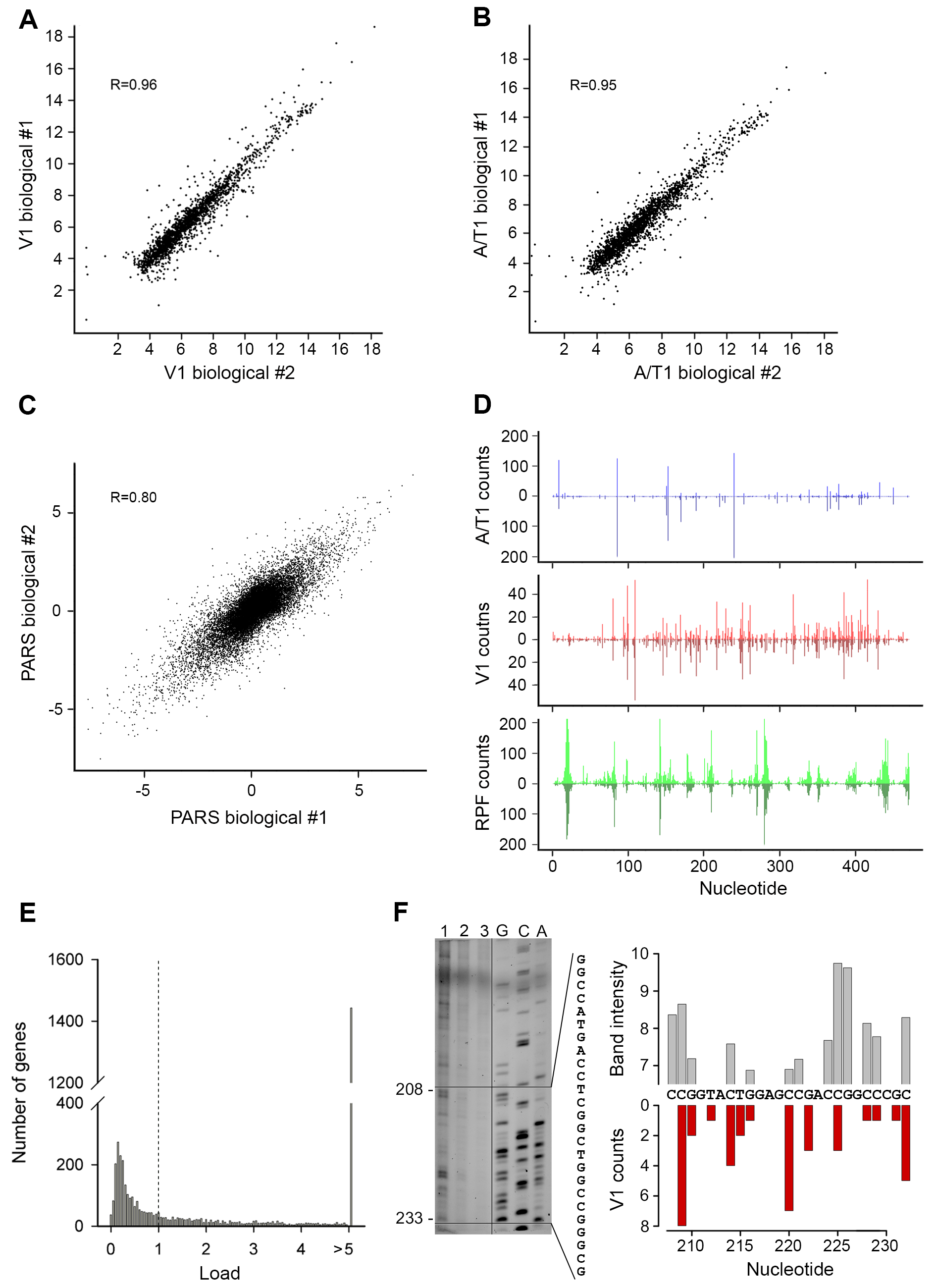

Supplement: S1 Fig — Pearson correlation between the log2 of read coverage for each transcript with load >1 (see panel E) digested by RNase V1 (A) or RNases A/T1 (B) in the two biological replicates. (C) Reproducibility of the PARS score for each nucleotide. To reduce the crowding in the plot, only 200000 randomly selected nucleotides were plotted. (D) Single gene example on the reproducibility of the various sequencing data. (E) Number of transcripts as a function of the transcript load [6], i.e. the PARS readouts from the merged biological replicates divided by the effective transcript length (that is the annotated transcript length minus the number of unmappable nucleotides). A threshold of 1 (vertical dashed line) was selected as also used previously for yeast PARS data [6]. (F) Footprint analysis of fluorescently-labeled ppiC mRNA digested with 0.05 U (lane 1) or 0.01 U (lane 2) RNase V1 compared to undigested mRNA (lane 3). The RNase V1-digestion pattern mirrors the V1 sequencing counts. The graphic insert represents an exemplary comparison between the intensity of the bands (gray bars) from designated area from the gel (horizontal lines between 207–234 nt) and the counts for the same gene obtained from the deep sequencing of the RNase V1 digested sample. The sequence derived from the Sanger sequencing (included next to the gel) is complementary to that in the plot. (TIF) [file pgen.1005613.s001.tif]

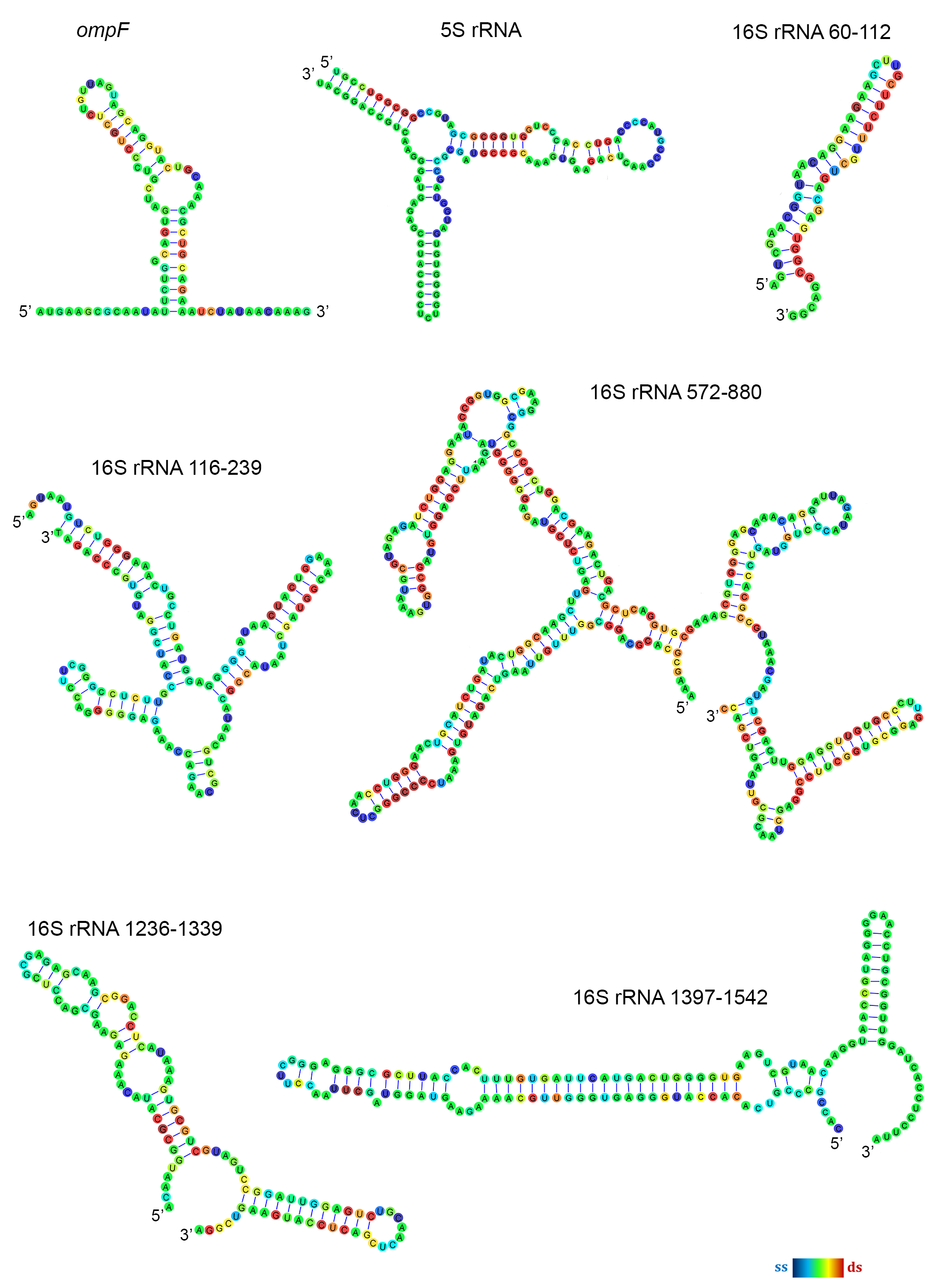

Supplement: S2 Fig — The PARS score was overlaid with the determined with OmpF [65], 5S rRNA [66] and 16S rRNA structure. The color intensity of the nucleotides reflects the PARS scores (rainbow legend). For more details on the PARS-based colorcoding see the legend to Fig 1B. For 16S rRNA, PARS score was overlaid with the determined structure. Solvent exposed helices were selected from the crystal structure [67,68] and overlaid with the experimentally determined PARS values. The solvent-exposed regions are cleaved first and this first phase of nucleolysis reports on the native structure allowing for more conservative PARS analysis. Nucleotides 60–107 –helix 6; nt 116–239 –helix 7 to 10; nt 572–880 –helix 20 to 26; nt 1236–133 –helices 41 and 42; nt 1397–1542–44 and 45. The color intensity of the 16S rRNA nucleotides reflects the magnitude of the PARS scores. (TIF) [file pgen.1005613.s002.tif]

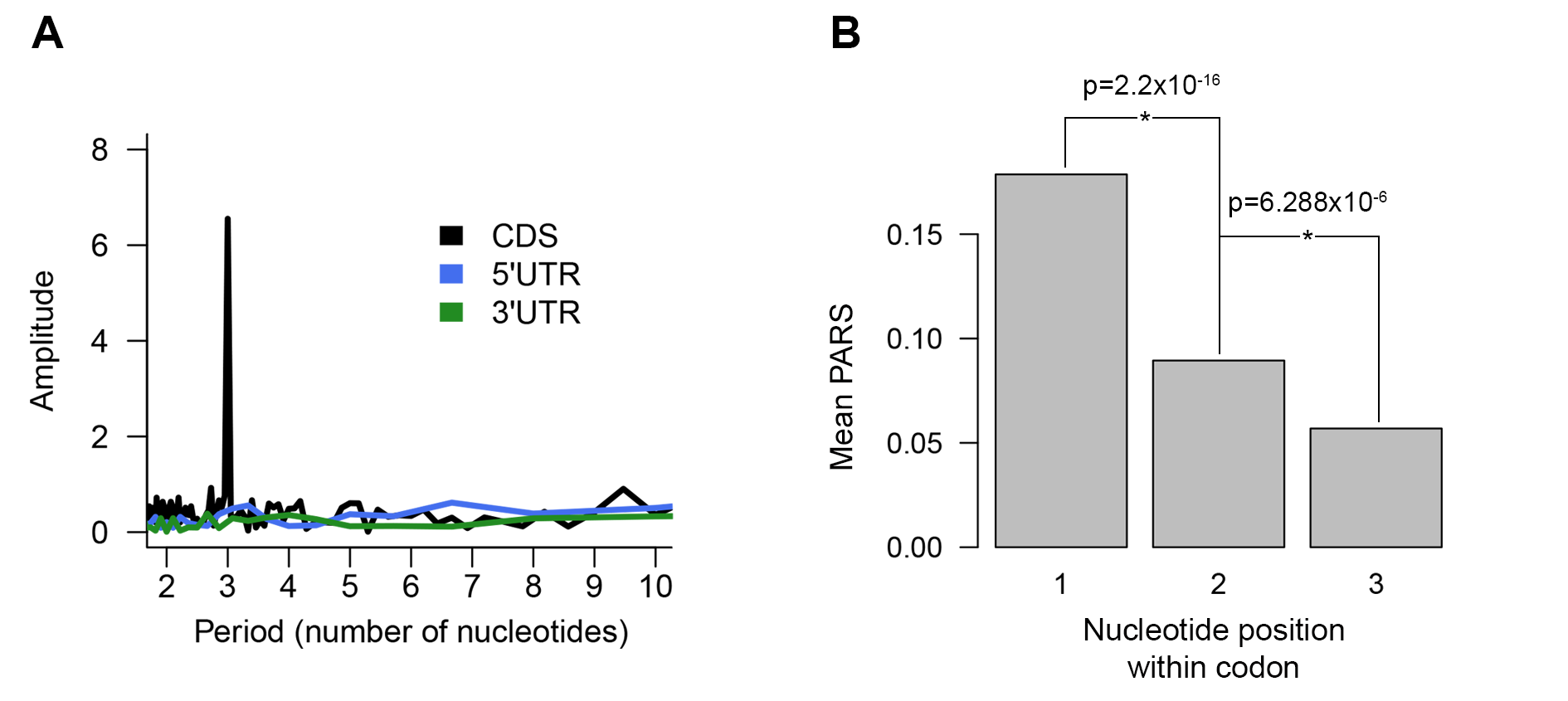

Supplement: S3 Fig — (A) Discrete Fourier transform analysis. Analyses were performed with the average PARS score over 10 to 99 nt downstream of the start codon, 99 to10 nt upstream of the stop codon for the CDSs, and 50 to 11 nt upstream of the start codon or downstream of the stop codon for the 5’UTR and 3’UTR, respectively. (B) Average PARS score for each of the three nucleotides of a codon, averaged across all codons. (TIF) [file pgen.1005613.s003.tif]

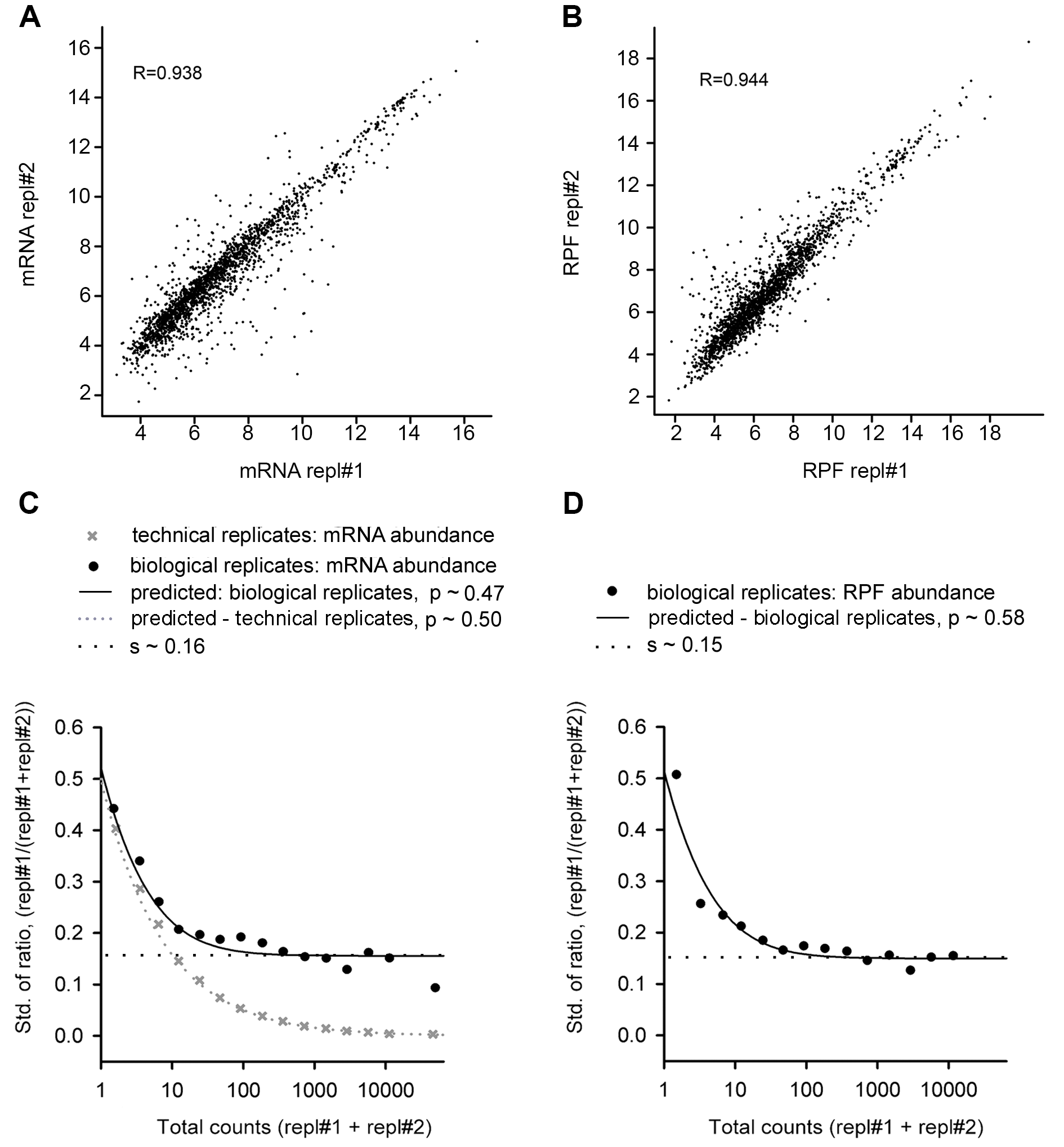

Supplement: S4 Fig — (A, B) Reproducibility of randomly fragmented mRNAs (A) and RPFs (B) of two biological replicates. The Pearson correlation coefficients, calculated between the log2 of the read coverage for each transcript with counts > 60 (see panel C, D) indicate that the RiboSeq and RNA-Seq analyses are highly reproducible. (C, D) Variability analysis of counting statistics on the error in quantification of RNA-Seq (C) and ribosome profiling (D). The two independent biological and technical mRNA (A) and RPF (B) replicates were used to estimate the biological variation compared to the technical one. The technical replicates are dominated by counting noise, thus s = 0 (Eq 1). A threshold of 120 total counts (i.e., 60 counts for each replicate) was chosen as for total reads >120 the variability approached the infinite-counts asymptote and the contribution of the counting statistics was little. For the RNA-Seq data set the fitting parameters are p = 0.47 and s = 0.16, and for the RPF data set are p = 0.58 and s = 0.15. By setting a threshold to 60 reads both in mRNA-Seq and RPF-analysis, the technical error is smaller than 5% of the biological variation. In total, 1.955 genes have >60 mRNA and RPF reads and have PARS over the selected threshold of 1 (S1 Fig). (TIF) [file pgen.1005613.s004.tif]

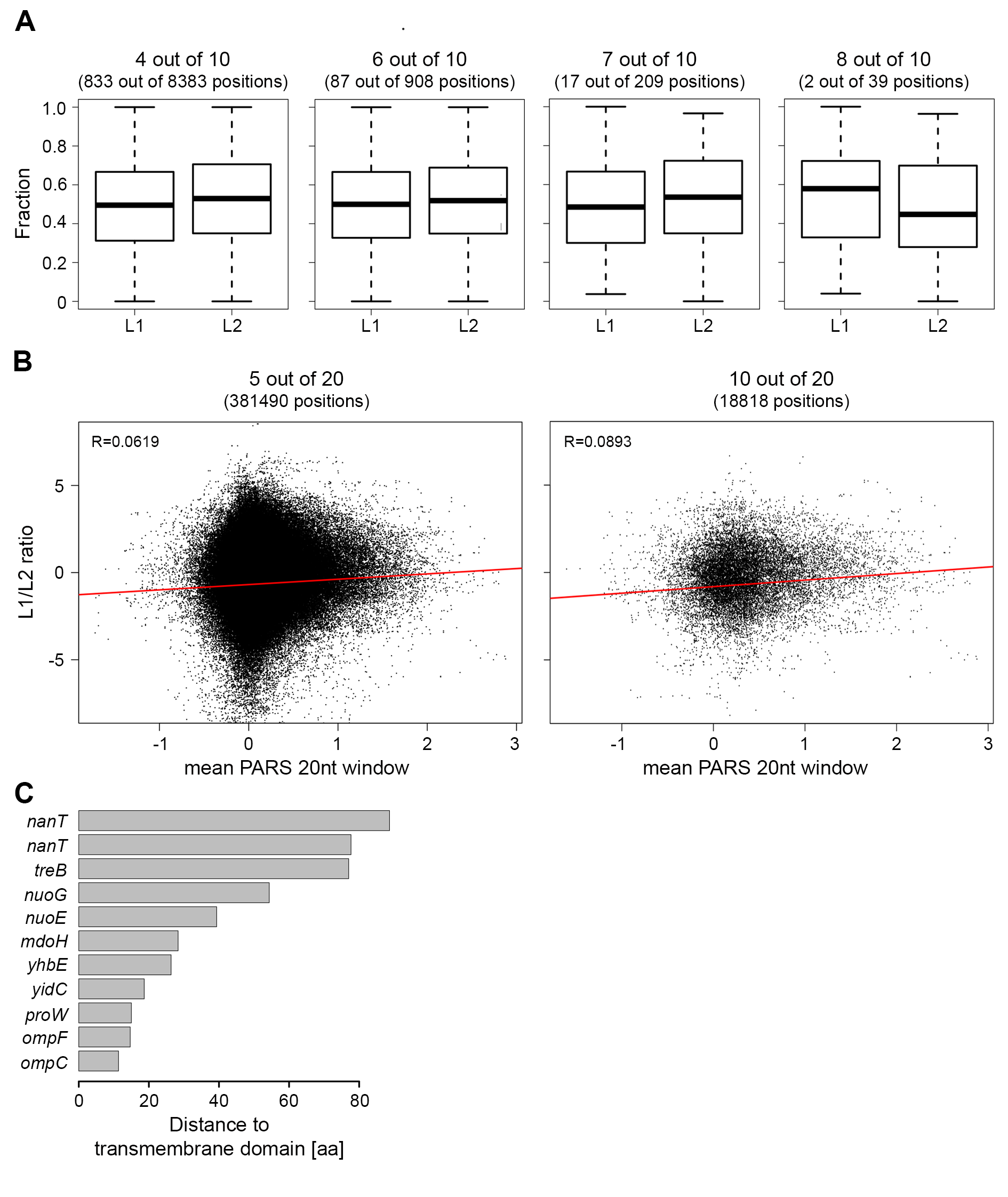

Supplement: S5 Fig — (A) The search is performed by varying the number of structured nucleotides (i.e. with positive PARS score) within a window of 10 nt. The numbers in brackets denote the number of 80th percentile positions within the whole set of detected structured positions. (B) The search is performed using the mean PARS score within a variable window (10 or 20 nt) under the restriction that within a window at least 5 nt (5 out of 10 nt or 5 out 20 nt) or 10 nt (10 out of 20 nt) have a PARS score different than zero. Note that this approach also cannot select for a minimal threshold PARS score over which the L1/L2 ratio becomes significant. PARS score gives the propensity of each nucleotide to partition between single or double stranded structure, therefore this propensity differs from the gain of energy which is determined by the type of nucleotide, the context and other factors. (C) Distance of the last residue of a transmembrane helix and the first nucleotide of a detected secondary structure which causes ribosomal stalling. The transmembrane helices of membrane proteins with structure-induced ribosome accumulation were predicted with www.cbs.dtu.dk/services/TMHMM/. Note that for nanT two structured regions were detected; the upper one reports on the structured region detected at 1234 nt. aa, amino acid. (TIF) [file pgen.1005613.s005.tif]

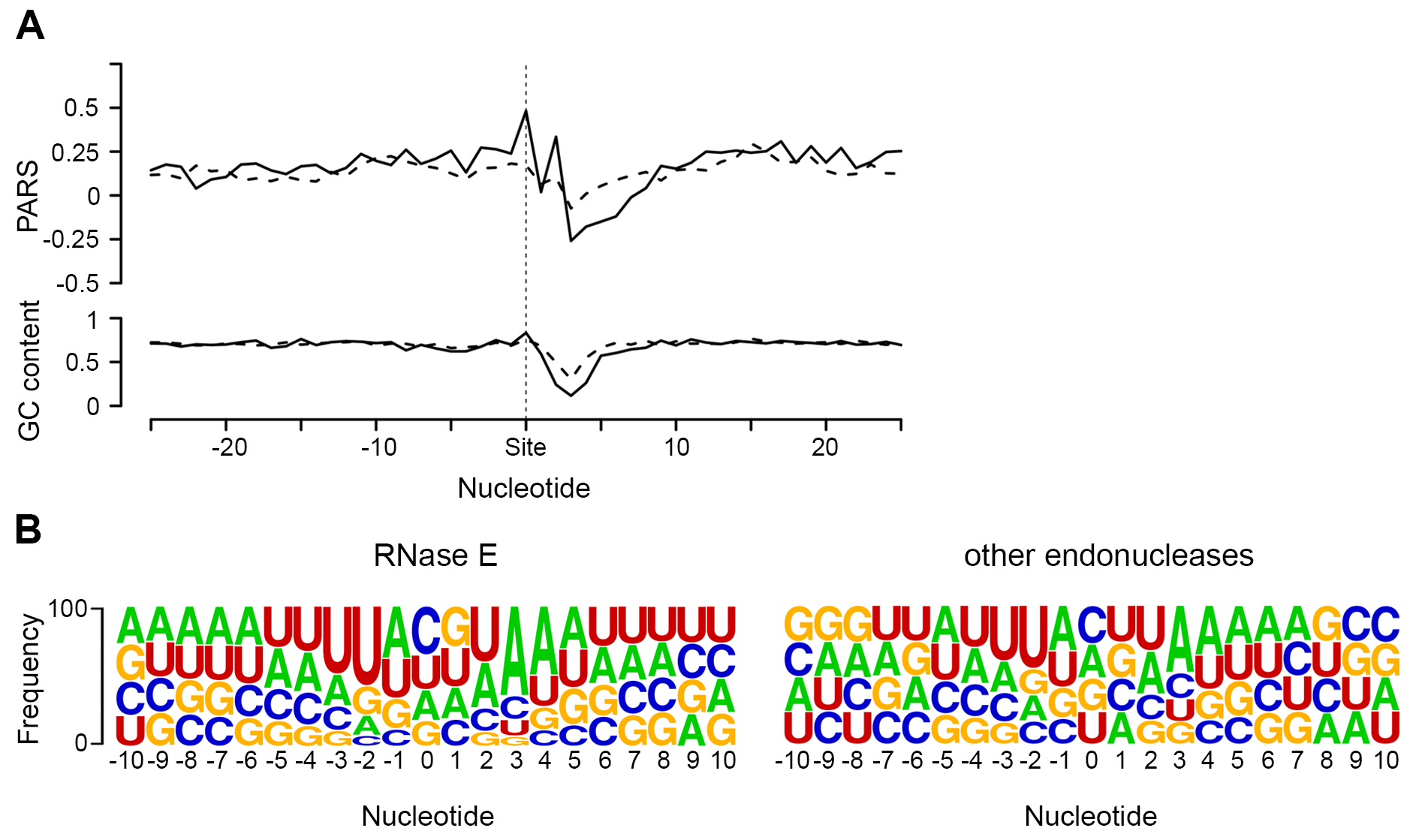

Supplement: S6 Fig — (A) The structural signature of the RNase E target sites differ significantly from that of other endonucleases (-8 to +2 nt, p = 0.0066, Mann-Whitney test). Average PARS score (top) and GC content (bottom) for each position around all identified ~1,800 RNase E cleavage sites (solid line) and additional ~5000 endonucleolytic sites (dashed line) detected under RNase E-depleted conditions [41]. (B) Frequency of the nucleotides around the RNase E cleavage site or other endonucleases whose PARS plot is shown in A. (TIF) [file pgen.1005613.s006.tif]

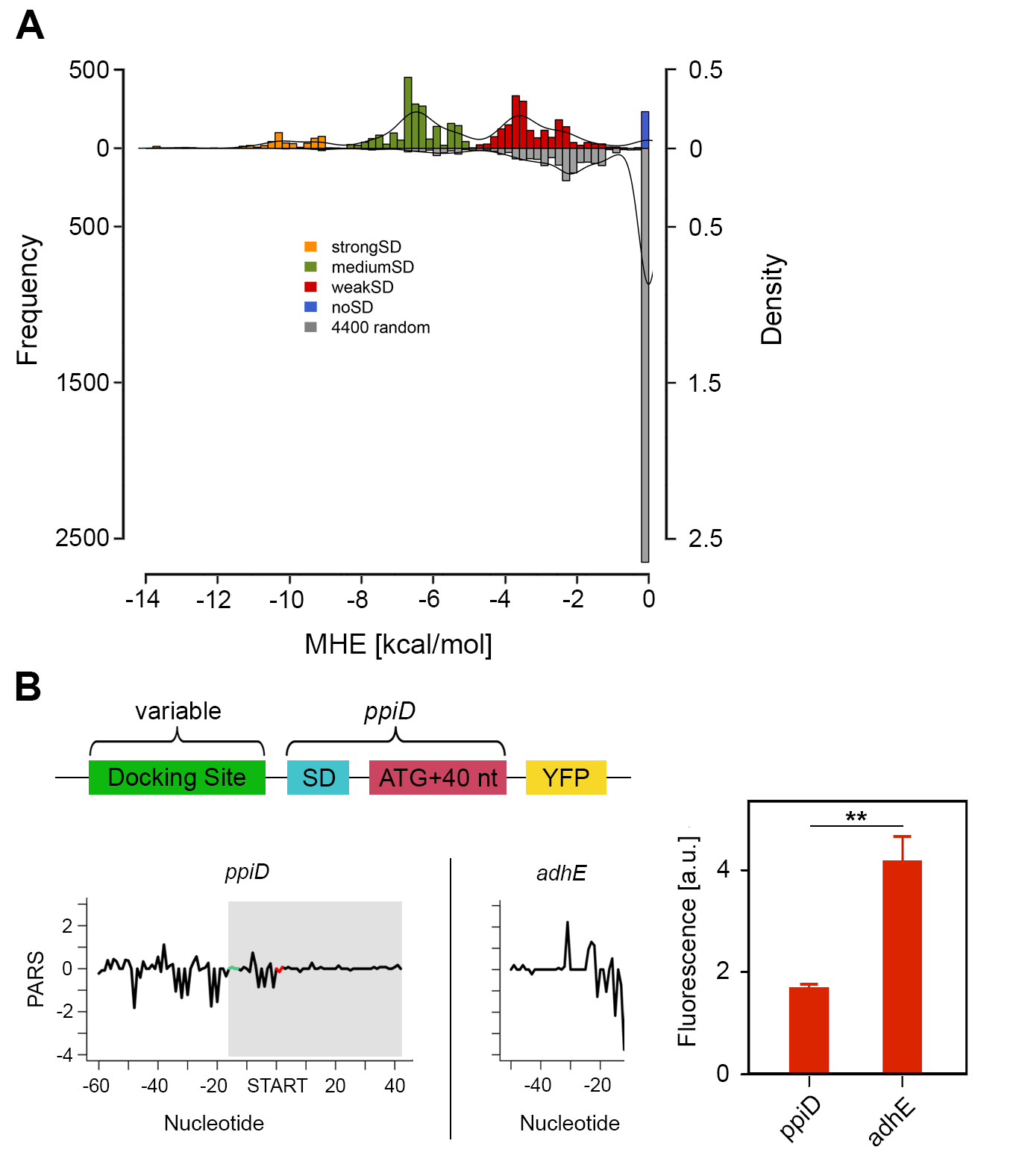

Supplement: S7 Fig — (A) Randomization of SD sequences. The MHE of paring randomized sequences (gray) with the anti-SD of the 16s rRNA is compared to the MHE distributions of naturally occurring SDs (Fig 4A). The fully randomized sample of all possible variations of randomized sequences of 8-nt length was ~65,000, however only 4,400 randomly chosen sequences (gray) are plotted to match the number of E. coli ORFs. The smoothed lines represent kernel density estimation (right y-axis). Color coding of the naturally occurring E. coli SD sequences is in Fig 4A. (B) FACS expression analysis of ppiD whose original sequence upstream of the SD (schematic) was replaced by that of adhE which has clearly different PARS score (adhE–-0.564, ppiD–-0.495). Data are means (n = 3) ± standard error of the mean (s.e.m.).**, P <0.01. (TIF) [file pgen.1005613.s007.tif]

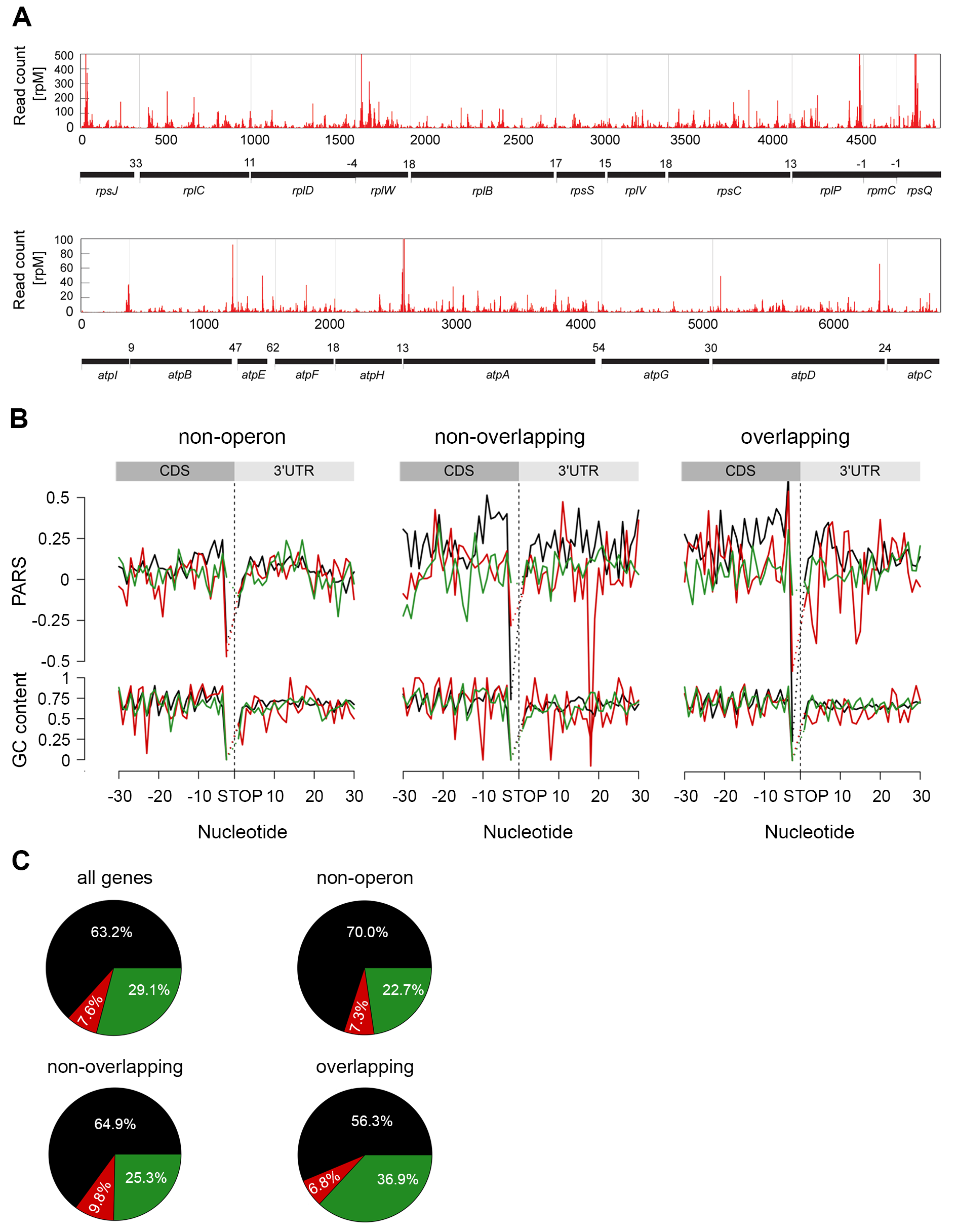

Supplement: S8 Fig — (A) Examples of genes organized in operons containing overlapping genes (upper panel) or non-overlapping (bottom panel) genes. RPF counts are plotted against the nucleotide position of operons. The gray vertical lines denote the boundaries of each ORF; the distance between the ORFs is given in nt in the schematic below the RPF-coverage profile. Negative numbers denote overlapping ORFs. (B) Average PARS score and GC content around the stop codon of different gene groups terminated with UAA (black), UAG (red) and UGA (green) stop codons. (C) Frequency of the three stop codons in different gene groups. UAA (black), UGA (green) and UAG (red). (TIF) [file pgen.1005613.s008.tif]
