# Supplementary material for: Secondary Structure across the Bacterial Transcriptome Reveals Versatile Roles in mRNA Regulation and Function
Source: PLoS Genet. 2015 Oct 23;11(10):e1005613. doi: 10.1371/journal.pgen.1005613 (PMC4619774; doi:10.1371/journal.pgen.1005613)
Supplement: S1 Table — Green, membrane proteins; blue, ribosomal proteins; orange, cytosolic proteins. (DOCX) [file pgen.1005613.s009.docx]

**Table S1. List of genes with identified ribosomal stalling induced by mRNA secondary structure.** Green, membrane proteins; blue, ribosomal proteins; orange, cytosolic proteins.

|  | | |
| --- | --- | --- |
| **Gene Name** | **Function** | **Detected pausing site** |
| ECK120000097.atpD | ATP synthase | 981 |
| ECK120000340.ftsY | SRP receptor | 1159 |
| ECK120000662.ompA | outer membrane protein A | 817 |
| ECK120000663.ompC | outer membrane protein C | 67 |
| ECK120000664.ompF | outer membrane protein C | 70 |
| ECK120000763.proW | proline ABC transporter - membrane subunit | 56 |
| ECK120001178.yidC | inner-membrane protein insertion factor | 341 |
| ECK120001455.yhbE | conserved inner membrane protein | 325 |
| ECK120001816.mdoH | membrane glycosyltransferase | 995 |
| ECK120001991.nuoE | NADH:ubiquinone oxidoreductase | 319 |
| ECK120001992.nuoG | NADH:ubiquinone oxidoreductase | 1514 |
| ECK120002029.treB | trehalose PTS permease | 1245 |
| ECK120002695.nanT | NanT sialic acid MFS transporter | 460, 1234 |
| ECK120002864.mscK | potassium dependent mechanosensitive channel MscK | 2293 |
| ECK120003892.bamB | Outer Membrane Protein Assembly Complex - BamB subunit | 231 |
| ECK120000394.glpT | glycerol-3-phosphate:phosphate antiporter | 1048 |
| ECK120000772.pssA | Phosphatidylserine synthase (component) | 233 |
| ECK120001020.trxA | thioredoxin 1 | 79 |
| ECK120004324.dcuS | DcuS sensory histidine kinase | 1244 |
| ECK120000353.fusA | elongation factor G | 1298, 1469 |
| ECK120000855.rplA | 50S ribosomal subunit protein L1 | 370 |
| ECK120000856.rplB | 50S ribosomal subunit protein L2 | 683 |
| ECK120000857.rplC | 50S ribosomal subunit protein L3 | 228 |
| ECK120000859.rplE | 50S ribosomal subunit protein L5 | 443 |
| ECK120000863.rplK | 50S ribosomal subunit protein L11 | 48 |
| ECK120000877.rpmB | 50S ribosomal subunit protein L28 | 38 |
| ECK120000892.rpsB | 30S ribosomal subunit protein S2 | 253 |
| ECK120000893.rpsC | 30S ribosomal subunit protein S3 | 432 |
| ECK120000898.rpsH | 30S ribosomal subunit protein S8 | 55 |
| ECK120000901.rpsK | 30S ribosomal subunit protein S11 | 365 |
| ECK120000902.rpsL | 30S ribosomal subunit protein S12 | 236 |
| ECK120001161.rimP | ribosome maturation protein | 306 |
| ECK120001768.typA | GTPase ribosome-associated | 524 |
| ECK120000131.carB | carbamoyl phosphate synthetase | 125, 258 |
| ECK120000209.deaD | DEAD-box RNA helicase | 1148 |
| ECK120000216.deoD | purine nucleoside phosphorylase | 38 |
| ECK120000351.fumC | fumarase C monomer | 197 |
| ECK120000387.glpD | glycerol-3-phosphate dehydrogenase subunit | 449 |
| ECK120000551.malK | maltose ABC transporter - ATP binding subunit | 1108 |
| ECK120000553.malP | maltodextrin phosphorylase monomer | 304 |
| ECK120000555.malT | MalT transcriptional activator | 644 |
| ECK120000630.nanA | N-acetylneuraminate lyase component | 587 |
| ECK120000695.pgk | phosphoglycerate kinase | 986 |
| ECK120000781.purA | Adenylosuccinate synthase (component) | 220, 1056 |
| ECK120000808.rbsD | ribose pyranase | 50 |
| ECK120000885.rpoB | RNA polymerase | 671, 1085, 1596, 2916 |
| ECK120000886.rpoC | RNA polymerase | 723 |
| ECK120000887.rpoD | RNA polymerase | 1565 |
| ECK120000970.sucC | succinyl-CoA synthetase | 359 |
| ECK120000994.tnaA | Tryptophanase | 908 |
| ECK120001032.tyrS | tyrosyl-tRNA synthetase | 224 |
| ECK120001056.valS | valyl-tRNA synthetase | 1832 |
| ECK120001401.plsX | fatty acid/phospholipid synthesis protein | 797 |
| ECK120001567.mfd | transcription-repair coupling factor | 2915 |
| ECK120001623.lptB | Lipopolysaccharide export ABC transporter ATP-binding protein | 49 |
| ECK120002177.gpmM | Phosphoglycerate mutase | 356 |
| ECK120002193.acnB | Aconitase B | 35 |
| ECK120002257.msrB | methionine sulfoxide reductase B | 304 |
| ECK120002445.pta | phosphate acetyltransferase | 1300 |
| ECK120002944.rnk | regulator of nucleoside diphosphate kinase | 312 |
| ECK120003088.rlmL | 23S rRNA m^2^G2445 methyltransferase | 31 |
| ECK120003163.nagZ | beta-N-Acetylglucosaminidase | 954 |
| ECK120000249.eco | ecotin monomer | 444 |
| ECK120000948.speA | arginine decarboxylase | 1166 |
