# Supplementary material for: Secondary Structure across the Bacterial Transcriptome Reveals Versatile Roles in mRNA Regulation and Function
Source: PLoS Genet. 2015 Oct 23;11(10):e1005613. doi: 10.1371/journal.pgen.1005613 (PMC4619774; doi:10.1371/journal.pgen.1005613)
Supplement: S2 Table — Positions denote the gene coordinates in the E. coli chromosome on either the forward (fwd) or reverse (rvs) strand. (DOCX) [file pgen.1005613.s010.docx]

**Table S2. List of the 64 RNase E cleavage positions**. Positions denotes the gene coordinates in the E. coli chromosome on either the forward (frw) or reverse (rvs) strand.

| **Gene** | **Position** | **Strand** | **Gene** | **Position** | **Strand** |
| --- | --- | --- | --- | --- | --- |
| *ftsI* | 93136 | fwd | *mglB* | 2237371 | rvs |
| *ftsI* | 93144 | fwd | *mglB* | 2238477 | rvs |
| *coaE* | 113122 | rvs | *nuoN* | 2388534 | rvs |
| *tsf* | 190802 | fwd | *nuoL* | 2392843 | rvs |
| *cyoC* | 447668 | rvs | *nuoL* | 2393046 | rvs |
| *cyoB* | 449135 | rvs | *nuoG* | 2397080 | rvs |
| *cyoB* | 449152 | rvs | *nuoG* | 2397802 | rvs |
| *yajG* | 452839 | rvs | *nuoG* | 2397961 | rvs |
| *uspG* | 640655 | rvs | *nuoC* | 2401345 | rvs |
| *fur* | 709635 | rvs | *nuoB* | 2402226 | rvs |
| *sdhC* | 754611 | fwd | *nuoB* | 2402633 | rvs |
| *sdhD* | 754977 | fwd | *nuoA* | 2403043 | rvs |
| *sdhD* | 755103 | fwd | *maeB* | 2576334 | rvs |
| *ybgF* | 778815 | fwd | *rnc* | 2701527 | rvs |
| *glnH* | 846518 | rvs | *pssA* | 2721316 | fwd |
| *ihfB* | 963323 | fwd | *rimM* | 2743400 | rvs |
| *ompF* | 985413 | rvs | *nlpD* | 2865691 | rvs |
| *ompF* | 985851 | rvs | *nlpD* | 2865699 | rvs |
| *ompF* | 985860 | rvs | *hybO* | 3144295 | rvs |
| *ompA* | 1018886 | rvs | *ispB* | 3331735 | fwd |
| *rne* | 1143045 | rvs | *smg* | 3430051 | rvs |
| *minD* | 1224121 | rvs | *rpsM* | 3440310 | rvs |
| *minD* | 1224577 | rvs | *envZ* | 3533575 | rvs |
| *oppB* | 1300973 | fwd | *malT* | 3551863 | fwd |
| *uspF* | 1433290 | rvs | *tnaA* | 3887308 | fwd |
| *aldA* | 1487678 | fwd | *tnaA* | 3887483 | fwd |
| *aldA* | 1487690 | fwd | *tnaA* | 3888162 | fwd |
| *manX* | 1900641 | fwd | *rbsC* | 3934182 | fwd |
| *cspC* | 1905439 | rvs | *fdhE* | 4079159 | rvs |
| *cspC* | 1905470 | rvs | *metL* | 4130357 | fwd |
| *rfbD* | 2109053 | rvs | *frdA* | 4379660 | rvs |
| *gatZ* | 2173243 | rvs | *ytfK* | 4437515 | fwd |
